# Comparative chloroplast genome analysis of *Sambucus* L. (Viburnaceae): inference for phylogenetic relationships among the closely related *Sambucus adnata* Wall. ex DC *Sambucus javanica* Blume

**DOI:** 10.3389/fpls.2023.1179510

**Published:** 2023-06-16

**Authors:** Emmanuel Nyongesa Waswa, Elijah Mbandi Mkala, Wyclif Ochieng Odago, Sara Getachew Amenu, Elizabeth Syowai Mutinda, Samuel Wamburu Muthui, Shi-Xiong Ding, Guang-Wan Hu, Qing-Feng Wang

**Affiliations:** ^1^ Key Laboratory of Plant Germplasm Enhancement and Specialty Agriculture, Wuhan Botanical Garden, Chinese Academy of Sciences, Wuhan, China; ^2^ Sino-Africa Joint Research Center, Chinese Academy of Sciences, Wuhan, China; ^3^ Botany Department, University of Chinese Academy of Sciences, Beijing, China

**Keywords:** chloroplast, genomes, phylogeny, taxonomy, *Sambucus*, Viburnaceae

## Abstract

*Sambucus* L. is found in the family Viburnaceae (syn. Adoxaceae) and encompasses approximately 29 accepted species. The complex morphology of these species has caused continued confusion concerning their nomenclature, classification, and identification. Despite previous attempts to resolve taxonomic complexities in the *Sambucus* genus, there are still unclear phylogenetic relationships among several species. In this study, the newly obtained plastome of *Sambucus williamsii* Hance. as well as the populations of *Sambucus canadensis* L., *Sambucus javanica* Blume, and *Sambucus adnata* Wall. ex DC were sequenced, and their sizes, structural similarity, gene order, gene number, and guanine–cytosine (GC) contents were analyzed. The phylogenetic analyses were conducted using the whole chloroplast genomes and protein-coding genes (PCGs). The findings revealed that the chloroplast genomes of *Sambucus* species exhibited typical quadripartite double-stranded DNA molecules. Their lengths ranged from 158,012 base pairs (bp) (*S. javanica*) to 158,716 bp (*S. canadensis* L). Each genome comprised a pair of inverted repeats (IRs), which separated the large single-copy (LSC) and small single-copy (SSC) regions. In addition, the plastomes contained 132 genes, encompassing 87 protein-coding, 37 tRNA, and four rRNA genes. In the simple sequence repeat (SSR) analysis, A/T mononucleotides had the highest proportion, with the most repetitive sequences observed in *S. williamsii*. The comparative genome analyses showed high similarities in structure, order, and gene contents. The hypervariable regions in the studied chloroplast genomes were *trnT-GGU*, *trnF-GAA*, *psaJ*, *trnL-UAG*, *ndhF*, and *ndhE*, which may be used as candidate barcodes for species discrimination in *Sambucus* genus. Phylogenetic analyses supported the monophyly of *Sambucus* and revealed the separation of *S. javanica* and *S. adnata* populations. *Sambucus chinensis* Lindl. was nested within *S. javanica* in the same clade, collaborating their conspecific treatment. These outcomes indicate that the chloroplast genome of *Sambucus* plants is a valuable genetic resource for resolving taxonomic discrepancies at the lower taxonomic levels and can be applied in molecular evolutionary studies.

## 1 Introduction

Chloroplasts (cp), the key organelles for photosynthesis and carbon fixation in green plants, are believed to have originated from ancestral cyanobacteria *via* endosymbiosis (Dyall et al., 2004). They are among the many distinguishing characteristic organelles in plant cells and possess genomes whose genetic information is maternally inherited from generation to generation (Birky, 1995). This genetic information contains enzymatic machinery essential for gene expression and encodes for many vital proteins that usually participate in photosynthesis and other metabolic processes (Green, 2011; Allen, 2015). The cp genomes are organized into large clusters of polycistronic transcribed genes that are highly conserved and comprise a single circular molecule with a quadripartite arrangement (Shen et al., 2018). A typical tetrad structure of the cp genome contains two copies of inverted repeat (IRa and IRb) regions and small single-copy (SSC) and large single-copy (LSC) regions in most plants (Wu et al., 2009; Kong and Yang, 2015). Plastomes’ IRs usually separate the LSC and SSC regions (Lee et al., 2007). The number of genes encoded by a circular cp genome is commonly 110–130, consisting of about 79 proteins, 30 transfer RNAs, and four ribosomal RNAse (Daniell et al., 2016).

Advances in high- throughput sequencing technologies have made large-scale cp genome sequence acquisition possible (Kyalo et al., 2020). Therefore, the cp genomes have highly been used as essential tools for comparative phylogenetic studies (Shen et al., 2018). For instance, numerical improvement of cp genome sequenced plants has been enhanced by the emergence and implementation of technical developments in DNA sequences such as next-generation sequencing (NGS) technologies. These approaches are cost-effective and time-efficient, enabling the exploration of high numbers of plant genomes at molecular levels (Raveendar et al., 2015). The cp genome analyses substantially contribute to the evolutionary and phylogenetic studies (Liu et al., 2013; Huang et al., 2014; Zhang et al., 2016; He et al., 2017) and have been applied at lower taxonomic levels to resolve close taxonomic relationships (Dong et al., 2012).

The family Viburnaceae as currently conceived encompasses the variously formerly classified genera in Caprifoliaceae, Sambucaceae, and Adoxaceae (Willis et al., 1973). Previous phylogenetic analyses within the family using morphological characters and *rbcL* sequences indicated *Adoxa* to be nested within *Sambucus* and inferred strong support for *Sambucus* monophyly (Chase et al., 1993; Olmstead et al., 1993). The internal transcribed spacer (ITS) regions of nuclear ribosomal DNA and morphological characters revealed *Adoxa* as a sister group to *Sambucus* (Eriksson and Donoghue, 1997). The close relatedness between *Sinadoxa*, *Tetradoxa*, and *Adoxa* was revealed by *rbcL* sequences and ITS regions (Eriksson and Donoghue, 1997; Jacobs et al., 2010). Moreover, a study on the phylogenetic relationship of Viburnaceae using the complete cp genomes confirmed a sister relationship between *Sambucus* and *Adoxa*–*Tetradoxa*–*Sinadoxa* groups (Ran et al., 2020).

**Figure 1:**
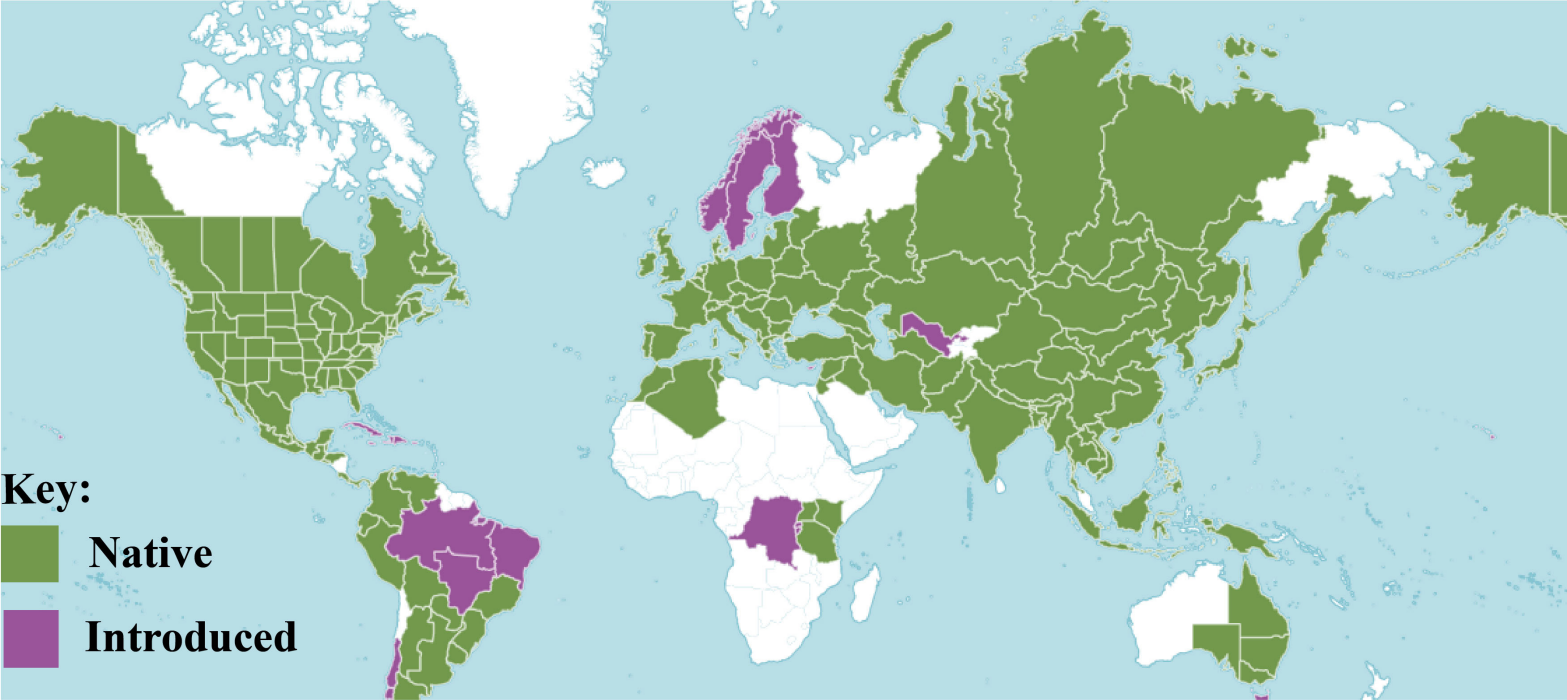
Distribution of *Sambucus* species (POWO, 2023).

*Sambucus* L. is a relatively small genus in the family Viburnaceae consisting of small trees, deciduous shrubs, and/or perennial herbs (Amini et al., 2019). They are highly distributed in the temperate and subtropical regions of the Northern Hemisphere with some species extending toward the Southern Hemisphere, surrounding both the high and low latitudes areas of North America, Asia, Europe, Northern Africa, West Indies, Eastern and South-eastern Australia, and the Andean region of South America **(** **Figure 1** **)**. The species of this genus are commonly known as elders or elderberries and are characterized by compound leaves (5–30 cm long) with serrated margins and five to nine pinnate to ovate-lanceolate or ovate-elliptic leaflets (Hummer et al., 2012). They form dense cymes of flowers that are white-yellowish or sometimes pinkish-purple. At maturity, elders bear brown-black, blue, red, orange, or yellow berry-like drupes (4–7 mm in diameter) (Fu et al., 2020).

*Sambucus* species have long been traditionally employed in the treatment of various ailments including bone fractures, diabetes, rheumatism, respiratory and pulmonary disorders, diarrhea, skin diseases, wounds, and inflammatory illnesses (Waswa et al., 2022). The species *Sambucus adnata* and *Sambucus williamsii* are by far the most popular Chinese traditional medicines extensively used by the Yi people of Liangshan in Sichuan province to treat bone fractures and rheumatism, whereas *Sambucus javanica* (syn. *Sambucus chinensis*) is medicinally employed to cure wounds and relieve throbbing pains (Wang et al., 2020; Otsuka et al., 2021). The medicinal properties of *Sambucus* plants are associated with the isolated bioactive metabolites such as phenolic compounds, terpenoids, fatty acids, and organic acids (Hearst et al., 2010; Do Nascimento et al., 2014; Przybylska-Balcerek et al., 2021). In addition, pharmacological investigations show that *Sambucus* plants are outstanding sources of antioxidants, antimicrobial, antidiabetic, anti-inflammatory, antidepressant, and anti-glycation activities, among others (Waswa et al., 2022).

Linnaeus was the first to provide the taxonomic description of the genus *Sambucus*, hence its botanical authority. The taxonomy of this genus is sophisticated due to plastic morphological characters induced by considerable diversity within species with vast geographical ranges and possible interspecific hybridizations (Applequist, 2015). von Schwerin (1920) recognized 28 species and several varieties, which relied heavily on important diagnostic morphological characters. At present, the most comprehensive taxonomic study was achieved by Bolli (1994), who included nine species in the genus by primarily focusing on morphological resemblances within the groups. Nevertheless, Bolli’s work lacked a molecular basis, and several researchers were hesitant to adopt it (Yatskievych, 2006; Applequist, 2015). In Bolli’s treatments, the two economically important members of the genus *Sambucus nigra* L. and *Sambucus canadensis* L. were given the subspecies status and recognized as *S. nigra* ssp. *nigra* (L.) R. Bolli and *S. nigra* ssp. *canadensis* (L.) R. Bolli. Conversely, molecular analysis of the microsatellites revealed their separation as distinct species (Clarke and Tobutt, 2006). Jacobs et al. (2009) assessed the phylogeny of *Sambucus* using the ITS, *trnK*, and *matK* molecular sets. The molecular datasets showed discrimination between the closely related *Sambucus racemosa* and *Sambucus ebulus*. Moreover, Amini et al. (2019) established delimitation of *S. nigra* and *S. ebulus* populations in the molecular investigation using micro-morphological characters and nuclear (nrDNA ITS) markers. Furthermore, the results from the complete cp genomes dataset displayed divergence between the populations of *S. nigra* and *S. williamsii* (Ran et al., 2020). Presently, the genus *Sambucus* encompasses 30 accepted taxa (22 species, four subspecies, and four varieties), 68 synonyms, and 73 ambiguous taxa **(** **Table S1** **)** (The Plant List, 2013; IPNI, 2023; POWO, 2023; WFO, 2023).

**Figure 2:**
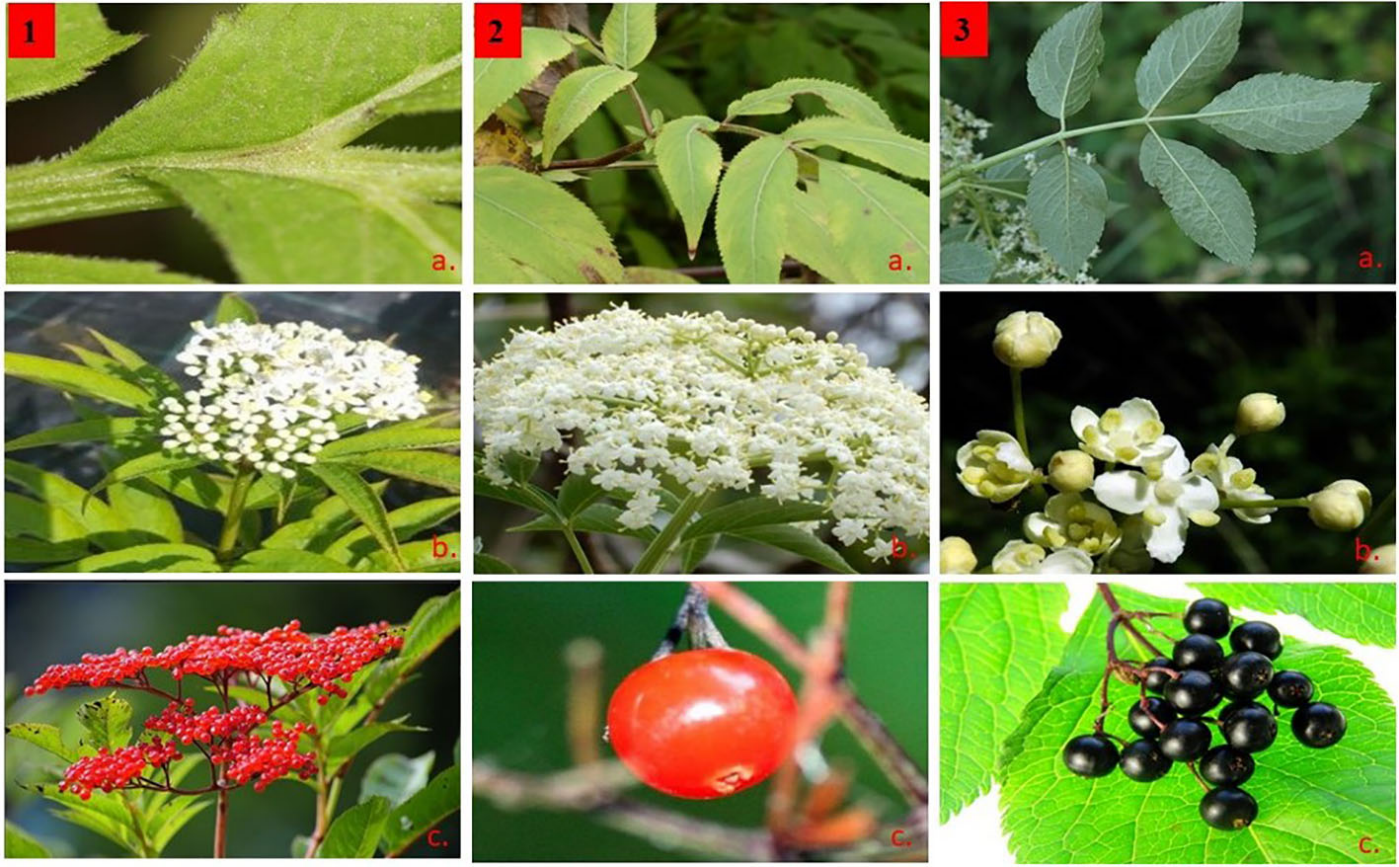
Morphological characters of *Sambucus* species. (1) *Sambucus adnata* Wall. ex DC. (2) *Sambucus javanica* Blume. (3) *Sambucus nigra* L. **(A)** Compound odd-pinnate leaves with serrated margins, **(B)** compound polychasial cyme, and **(C)** small rounded berry-like drupes.

Representatives of the genus *Sambucus* usually show high variability within individual species with wide geographical ranges, and thus artificial classification based solely on morphological trait-based systems is undependable and controversial. Thus, the outcome of this classification cannot be outrightly adopted and require molecular-based studies to confirm their taxonomic treatments. Elucidating the phylogenetic relationships of *Sambucus* plants using complete plastomes is essential in understanding their taxonomic treatments. In our fieldwork, several species of *Sambucus* were collected from China including populations of *S. adnata* and *S. javanica* that were observed to share high morphological similarities, including bladelike stipule leaves, stems with white pith, pedunculate inflorescences, calyx urceolate, and red fruits (http://www.efloras.org/). They exhibited limited phenotypic variations (**Figure 2**), necessitating investigations of their molecular affinities and phylogenetic relationships. The results obtained by Ran et al. (2020) using a complete chloroplast genome sequence dataset indicated that *S. javanica* Blume is a close relative of *S. adnata* Wall. ex DC. However, the molecular investigation attempts to assess species limits between the closely related *S. adnata* and *S. javanica* are lacking. The present study aimed to I) characterize and compare the cp genomes of *S. williamsii* and the populations of *S. adnata*, *S. javanica*, and *S. canadensis*; II) examine the phylogenetic relationships of the main clades of Viburnaceae, with a particular focus on the generic status of *Sambucus*; III) perform comparative analyses of *Sambucus* for future species identification and phylogeographic studies; IV) e xplore the taxonomic treatment of *S. adnata* Wall. ex DC and *S. javanica* Blume.

## 2 Materials and methods

We performed the comparative genomics and phylogenetic relationships of various *Sambucus* species obtained from Africa and Asia. In this investigation, the complete chloroplast genomes of *S. canadensis* were sequenced and reported for the first time.

### 2.1 Plant material collections

Fresh leaf materials of *Sambucus* plants were obtained from several areas in China and Kenya **(** **Table S2** **)**. The samples that did not show any obvious disease symptoms were washed and dried in silica gel for preservation until DNA extraction. Voucher specimens were deposited and stored in the Herbarium of Wuhan Botanical Garden (HIB) at −80°C pending chloroplast DNA extraction.

### 2.2 Plant DNA extraction, sequencing, and assembly

The total genomic DNA of each specimen was extracted from 100 mg of the leaves using improved cetyltrimethylammonium bromide (CTAB) protocol (Doyle, 1990). The NanoDrop spectrometer (Beckman Coulter, Krefeld, Germany) and gel electrophoresis (Beijing Liuyi Instrument Factory, Beijing, China) were used to determine the quantity and quality of the isolated DNA material. The other 30 species from the family Viburnaceae and two outgroups were downloaded from the NCBI (https://www.ncbi.nlm.nih.gov/) (**Table S2**). The cp genomes were sequenced using the Illumina platform at Novo Gene Company (Beijing, China). High-quality reads were used for *de novo* assembly to reconstruct the *Sambucus* chloroplast genome using GetOrganelle v.1.7.2 with a word size of 150 and K-mer size of 105 using plastome data of *S. chinensis* (MW455170) as a reference. The resulting scaffolds and their connectivity were visualized using Bandage 0.7.1 software (Wick et al., 2015) to authenticate the produced plastid genomes. Finally, the quality of the assembled plastomes was confirmed based on the reading level by aligning the trimmed raw reads to the *de novo* assemblies using Geneious Prime 2021 (Kearse et al., 2012), with medium-to low-sensitivity option and iteration up to five times (Hourahine et al., 2020). **File S1** contains the text generated following the assembly of the complete cp genome of *Sambucus*, while its depiction is illustrated in **Figure S1**.

### 2.3 Annotation of the chloroplast genomes

The complete cp genome sequences were annotated using CPGAVAS2 software (Shi et al., 2019), with the gene boundaries (intron/exon) and codons (start/stop) checked and modified manually using the reference genome. tRNAscan-SE v1.21 software (Meier-Kolthoff et al., 2013) was used with its default settings to confirm the tRNAs. The web application (https://irscope.shinyapps.io/chloroplot/) was used to create the gene maps of *Sambucus* cp genomes. The complete cp genomes were deposited at the GenBank with the following accession numbers: *S. javanica* (OM868260, ON006397, ON006398, and ON006402), *S. williamsii* (OM937121), *S. canadensis* (OM937119 and OM937120), and *S. adnata* (ON006399, ON006400, and ON006401).

### 2.4 Repeats and codon usage analysis

The whole cp genomes of *Sambucus* species were aligned in GENEIOUS v11.1.4 (Kearse et al., 2012), using MAFFT multiple aligner v7. The Perl script MISA (Beier et al., 2017) was used to identify the simple sequence repeat (SSR) loci (i.e., mono-, di-, tri-, tetra-, penta-, and hexa-) nucleotide repeats with the following thresholds: 10 repeats for mononucleotides, five repeats for dinucleotides, four repeats for trinucleotides, and three repeats each for tetranucleotides, pentanucleotides, and hexa nucleotides. In addition, the number of positions of repeat elements was estimated using the program REputer (Kurtz et al., 2001), including forward, palindromic, complementary, and reverse repeats with a minimum size of 30 bp and sequencing identity of not less than 90% with the hamming distance of 3.

### 2.5 Comparative complete chloroplast genome analysis

The boundary shifts of *Sambucus* plastomes at the IR borders were examined using the IRscope online program (https://irscope.shinyapps.io/irapp/). For expansion/contraction analysis, 10 plastomes were compared with the *S. chinensis*_MW455170 plastome using Geneious v11.1.4 (Kearse et al., 2012). Afterward, the disparities of the gene position at the plastome boundary (IR-SC) of the four junctions (JLB–LSC/IRB, JSB–IRB/SSC, JSA–SSC/IRA, and JLA–IRA/LSC) were assessed. The 10 plastomes and the reference were aligned using the progressive Mauve algorithm in default settings (Jin et al., 2020) to detect the gene inversions. The parameters were set to automatically calculate the seed weight (15) and locally collinear blocks (LCBs) with the minimum LCB score of 30,000 (Darling et al., 2004). The cp genomes of the studied species were constructed using the mVISTA program (Frazer et al., 2004) in Shuffle-LAGAN mode, with *S. chinensis*_MW455170 as a reference to assess divergence in the genomic structures. Sliding windows analysis in Dnasp v5.10 (Librado and Rozas, 2009) was used to calculate the nucleotide sequence variability. The window length was calibrated at 600 bp with a 200- bp step size.

### 2.6 Synonymous and non-synonymous substitution rates

We also assessed the substitutions of synonymous (Ka) and non-synonymous (Ks) ratios (Ka/Ks) using the KaKs calculator (Zhang et al., 2006). In this analysis, the protein-coding sequences were extracted using PhyloSuite (Zhang et al., 2020), and sequence alignment was performed using MAFFT v7 software (Katoh and Standley, 2013). Unrealistic Ka/Ks ratios were excluded to ensure precise screening of conserved and divergent genes. Thus, we adopted a more accurate threshold screening: Ka/Ks < 0.01 treated as qualified conserved genes, Ka/Ks = 1 for neutral selection, and Ka/Ks >1 (greater than 1) as positively selected orthologs based on Abdullah et al. (2020) and Ka/Ks < 1 (less than 1) for purifying selection.

### 2.7 Phylogeny

The phylogenies within the studied family were examined using the maximum-likelihood (ML), Bayesian inference (BI), and Neighbor-Net (NN) methods. To infer phylogenetic relationships in the family Viburnaceae, 42 species were examined that incorporated the newly obtained plastomes of *Sambucus* species, including *S. williamsii* (OM937121) and populations of *S. javanica* (OM868260, ON006397, ON006398, and ON006402), *S. adnata* (ON006399 and ON006400), and *S. canadensis* (OM937119 and OM937120) **(** **Table S2** **)**. Additionally, the complete cp genome sequences of five other *Sambucus* species, together with 22 *Viburnum* and three *Adoxa* species (including *Adoxa moschatellina*, *Adoxa corydalifolia* (*Sinadoxa corydalifolia*), and *Adoxa omeiensis* (*Tetradoxa omeiensis*)) and two outgroups (*Panax ginseng* (MH049735) and *Eleutherococcus gracilistylus* (KT153020)) from Araliaceae family were downloaded from GenBank **(** **Table S2** **)**.

The downloaded plastomes that contained errors were re-annotated. Protein-coding genes were extracted using PhyloSuite v 1.2.2 software (Zhang et al., 2020), and the “–auto” strategy and normal alignment mode were used to align the sequences in MAFFT v 7 (Katoh and Standley, 2013). The “automated1” command was set to omit gap sites using trimAl (Capella-Gutiérrez et al., 2009), while the fragments that were aligned ambiguously were eliminated by default parameter settings in Gblocks (Talavera and Castresana, 2007). The cleaned sequences were concatenated into different formats for other analyses. By applying the corrected Akaike information criterion (AICc) criterion and all algorithms, pre-defined partitions were chosen by the best partitioning arrangement and evolutionary models in PartitionFinder2 (Lanfear et al., 2017). Based on our obtained dataset of 58 protein-coding genes that were common in all species, we used ML phylogenies embedded in IQtree (Nguyen et al., 2015) with the GTR+R2+F model for 5000 ultrafast and the BI by Bayes (Ronquist and Huelsenbeck, 2003) to perform the phylogenetic analysis. The maximum-likelihood phylogeny was inferred based on the Minh et al. (2013) model and the likelihood-ratio test defined by Guindon et al. (2010). The complete cp genome data were aligned using clustal format by MAFFT (v7.504). In this study, we used the term “clade” to refer to clusters for the recovered phylogenies. Moreover, we adopted the name “Adoxoideae” to represent the *Sambucus*–*Adoxa*–*Tetradoxa*–*Sinadoxa* group and “Adoxina” for the *Adoxa*–*Tetradoxa*–*Sinadoxa* (Adoxina) group based on Jacobs et al. (2010). The distance-based network construction method NN incorporated in the SplitsTree4 v.4.14.4 (Huson and Bryant, 2001) was used to reconstruct phylogenetic split networks (Levy and Pachter, 2011). We used the term “lineage” to denote a group of specimens in the split graph (Neighbor-Net diagram).

## 3 Results

### 3.1 General characterization of chloroplast genomes

**Figure 3:**
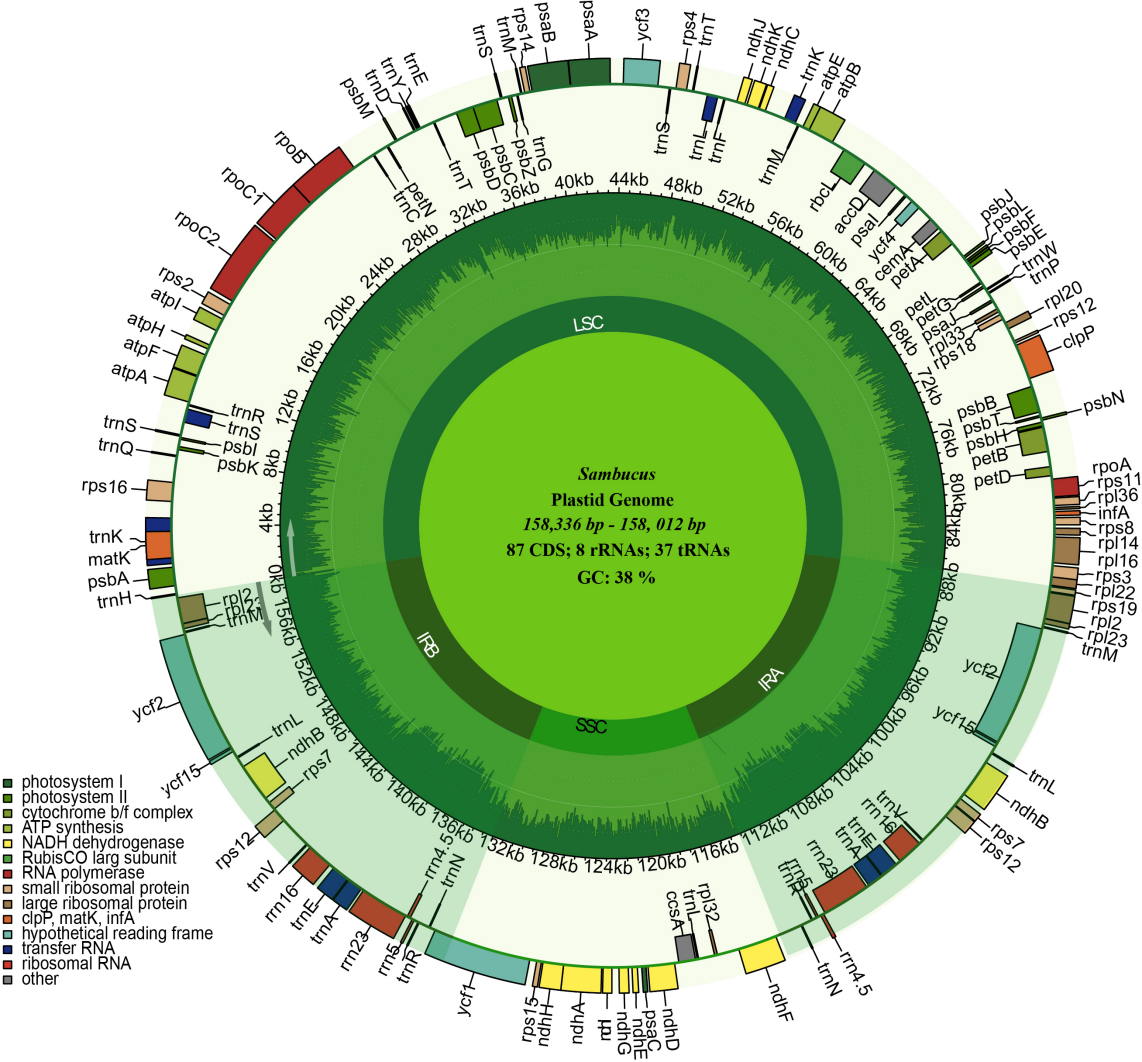
Circular maps of the chloroplast genomes of three *Sambucus* species. Genes outside the circle are transcribed clockwise, while the genes inside the circle are transcribed counterclockwise. The dark gray plot in the inner circle corresponds to the guanine–cytosine (GC) content. LSC, SSC, and IRs (IRA and IRB) denote large single- copy, small single- copy, and inverted repeat regions, respectively.

The assembled and annotated genomes varied in size (158,012–158,716 bp) and displayed a quadripartite conformation. They contained two copies of IR regions ranging from 26,149 to 26,269 bp. The IRs were segregated by the SSC and LSC regions, which varied in size from 18,920 to 19,099 and 86,624 to 87,376 bp, respectively **(** **Figure 3**; **Table 1** **)**. The genomes showed high similarities in guanine–cytosine (GC) contents of 38.0%, with the LSC, SSC, and IR regions exhibiting respective values of 36.3%, 31.7%, and 43.0% **(** **Table 1** **)**.

**Table 1:** Detailed comparison of the complete chloroplast genomes of *Sambucus* species.

	*Sambucus javanica*				*Sambucus canadensis*		*Sambucus williamsii*	*Sambucus adnata*		
Accession	OM868260	ON006397	ON006398	ON006402	OM937120	OM937119	OM937121	ON006401	ON006400	ON006399
**Genome size (bp)**	158,716	158,728	158,772	158,705	**158,012**	**158,012**	158,336	**158,573**	**158,595**	**158,571**
**LSC length (bp)**	87,376	87,374	87,375	87,366	**86,624**	**86,624**	86,768	**87,327**	**87,347**	**87,289**
**SSC length (bp)**	19,042	19,056	19,099	19,041	**18,962**	**18,962**	19,030	**18,920**	**18,922**	**18,940**
**IR length (bp)**	26,149	26,149	26,213	26,149	**26,313**	**26,213**	26,269	**26,163**	**26,163**	**26,149**
**LSC GC content %**	36.3	36.3	36.3	36.3	**36.3**	**36.3**	36.3	**36.3**	**36.3**	**36.3**
**SSC GC content %**	31.9	31.9	31.9	31.9	**31.8**	**31.8**	31.6	**32.0**	**32.0**	**32.0**
**IR GC content %**	43.0	43.0	43.0	43.0	**43.0**	**43.0**	43.0	**43.0**	**43.0**	**43.0**
**Total GC content %**	38.0	38.0	38.0	38.0	**38.0**	**38.0**	38.0	**38.0**	**38.0**	**38.0**
**No. of genes**	132	132	132	132	**132**	**132**	132	**132**	**132**	**132**
**No. of PCGs**	87	87	87	87	**87**	**87**	87	**87**	**87**	**87**
**No. of rRNAs**	8	8	8	8	**8**	**8**	8	**8**	**8**	**8**
**No. of tRNAs**	37	37	37	37	**37**	**37**	37	**37**	**37**	**37**
**Duplicated genes**	19	19	19	19	**19**	**19**	19	**19**	**19**	**19**

GC, guanine–cytosine; IR, inverted repeat; LSC, large single copy; PCG, protein-coding gene; SSC, small single copy.

The bold values show segregation of one population from the other.

We detected 132 functional genes encompassing 87 protein-coding, 37 tRNA, and eight rRNA genes. Among them, 19 genes were replicated in the IR regions, comprising seven protein-coding genes (PCGs) (*ycf1*, *rpl2*, *rps7*, *ndhB*, *ycf2*, *rpl23*, and *rps12*), four rRNA genes (*rrn5*, *rrn4.5*, *rrn23*, and *rrn16*), and eight tRNA genes (*trnN-GUU*, *trnR-ACG*, *trnA-UGC*, *trnL-GAU*, *trnV-GAC*, *trnL-CAA*, *trnI- CAU*, and *trnH-GUG*) **(** **Figure 3** **)**. *rps12*, *clpP*, and *ycf3* genes were identified to have two introns, whereas 15 genes including *trnK-UUU*, *trnG-UCC*, *trnL-UAA*, *trnV-UAC*, *trnl-GAU*, *trnA-UGC*, *petB*, *petD*, *atpF*, *ndhB*, *ndhA*, *rpl1*, *rpl2*, *rps16*, and *rpoC1* contained one intron. *ycf1* genes were detected as pseudogenes in the studied *Sambucus* species with a trans-splicing event, whereby the 5′ end of *rps12* gene located in the LSC part and the 3′ situated at the IRa/IRb region were observed. Comparison between the studied and other selected cp genomes showed no significant gene order rearrangements or inversions. The genes encoded by *Sambucus* cp genomes are listed in **Table 2**.

**Table 2:** Genes existing in the chloroplast genome of *Sambucus*.

Gene category	Group of genes	Gene name	Number
RNA genes	Ribosomal RNAs	*rrn16*(×2), *rrn23(×2)*, *rrn4.5(×2)*, *rrn*5(×2) *trnH-GUG*, *trnK-UUU* ^±^, *trnQ-UUG*, *trnS-GCU*, *trnG-UCC^±^*, *trnR-UCU trnC-GCA*, *trnD-GUC*, *trnY-GUA*, *trnE-UUC*, *trnT-GGU trnS-UGA*, *trnG-UCC*, *trnfM-CAU*, *trnS-GGA*, *trnT-UGU*	8
	Transfer RNAs	*trnL-UAA^±^*, *trnF-GAA*, *trnV-UAC^±^*, *trnM-CAU*, *trnW-CCA trnP-UGG*, *trnl-CAU (×2)*, *trnL-CAA (×2)*, *trnV-GAC (×2)* *trnl-GAU^±^ (×2)*, *trnA-UGC^±^ (×2)*, *trnR-ACG (×2)*, *trnN-GUU (×2) trnL-UAG*, *trnM-CAU*, *trnT-GGU*, *trnP-GGG*	37
Photosynthetic genes	Photosystem I	*psaB*, *psaA*, *psal*, *psaJ*, *psaC*	5
	Photosystem II	*psbA*, *psbB*, *psbC*, *psbD*, *psbE*, *psbF*, *psbI*, *psbJ*, *psbK*, *psbL*, *psbM*, *psbN*, *psbT*, *psbZ*, *ycf3*	15
	Cytochrome	*petN*, *petA*, *petL*, *petG*, *petB^±^*, *petD^±^*	6
	Subunits of ATP synthase	*atpA*, *atpB*, *atpE*, *atpF^±^*, *atpH*, *atpI*	6
	Rubisco	*rbcL*	1
	NADH dehydrogenase subunits	*ndhJ*, *ndhK*, *ndhC*, *ndhB^±^ (×2)*, *ndhD*, *ndhE*, *ndhG* *ndhl*, *ndhA^±^*, *ndhF (x2)*, *ndhH*	12
Self-replication	Ribosomal proteins (large units)	*rpl33*, *rpl20*, *rpl36*, *rpl14*, *rpl16^±^*, *rpl22 z*, *rpl2^±^ (×2)*, *rpl23(×2)*, *rpl32*	11
	Ribosomal proteins (small units)	*rps16^±^*, *rps2*, *rps14*, *rps4*, *rps18*, *rps12^γ^ (×2)*, *rps11*, *rps8*, *rps7(×2)*, *rps15*, *rps3*, *rps19*	14
	RNA polymerase	*rpoC2*, *rpoC1^±^*, *rpoB*, *rpoA*	4
Other proteins		*accD*, *cemA*, *infA*, *ccsA*, *matK*, *clpP^γ^*	6
Genes of unknown functions	Hypothetical proteins and conserved reading frame	*ycf5 (x2)*, *ycf4*, *ycf3^γ^*, *ycf2(×2)*, *ycf1* Ψ	7
Total			132

(×2) denotes duplicated genes in the IR regions. Ψ indicates pseudogenes.

^±^Genes with a single intron.

^γ^Genes with two introns.

### 3.2 Repeat sequence characterization

**Figure 4:**
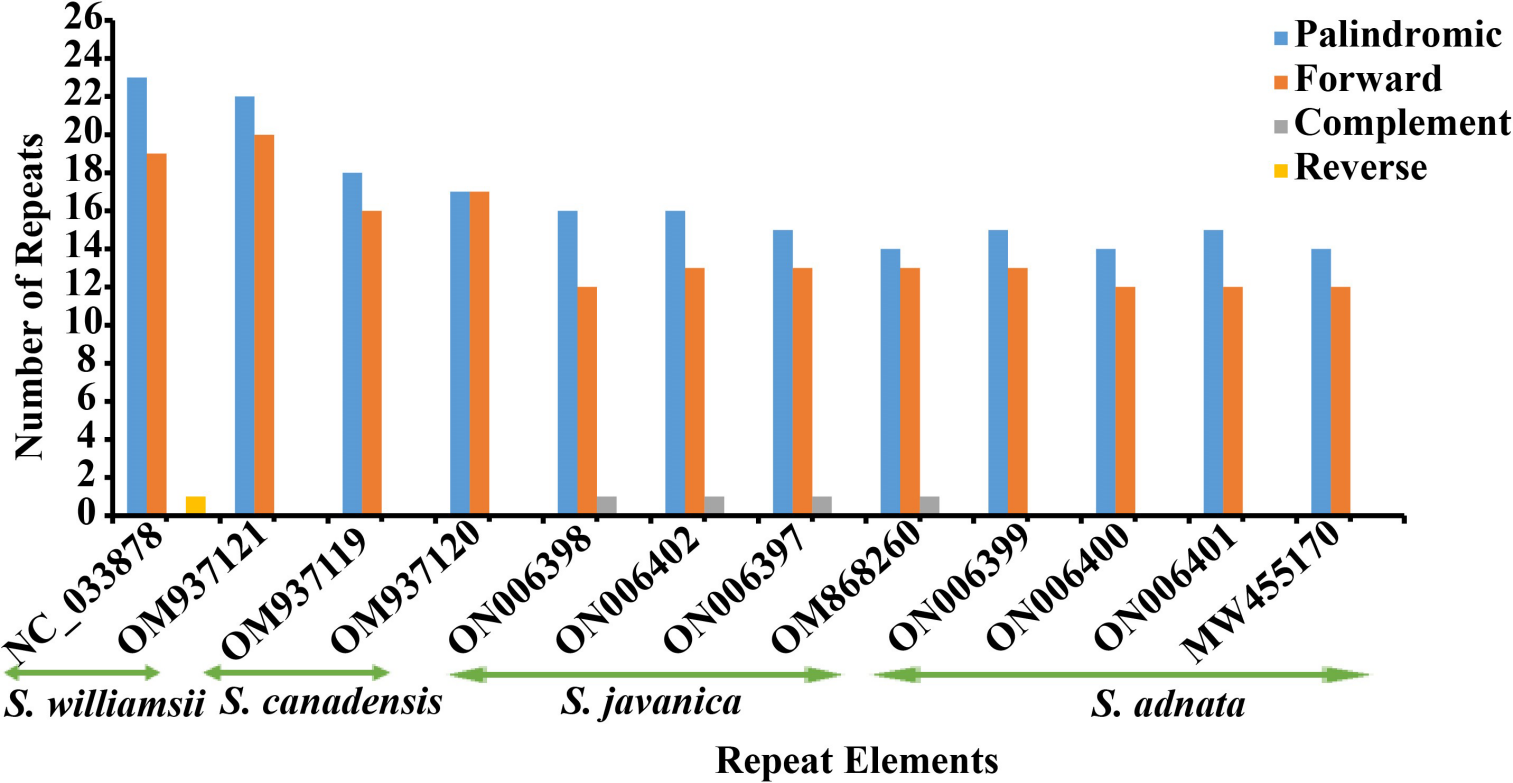
Long repeat elements in *Sambucus* species.

**Figure 5:**
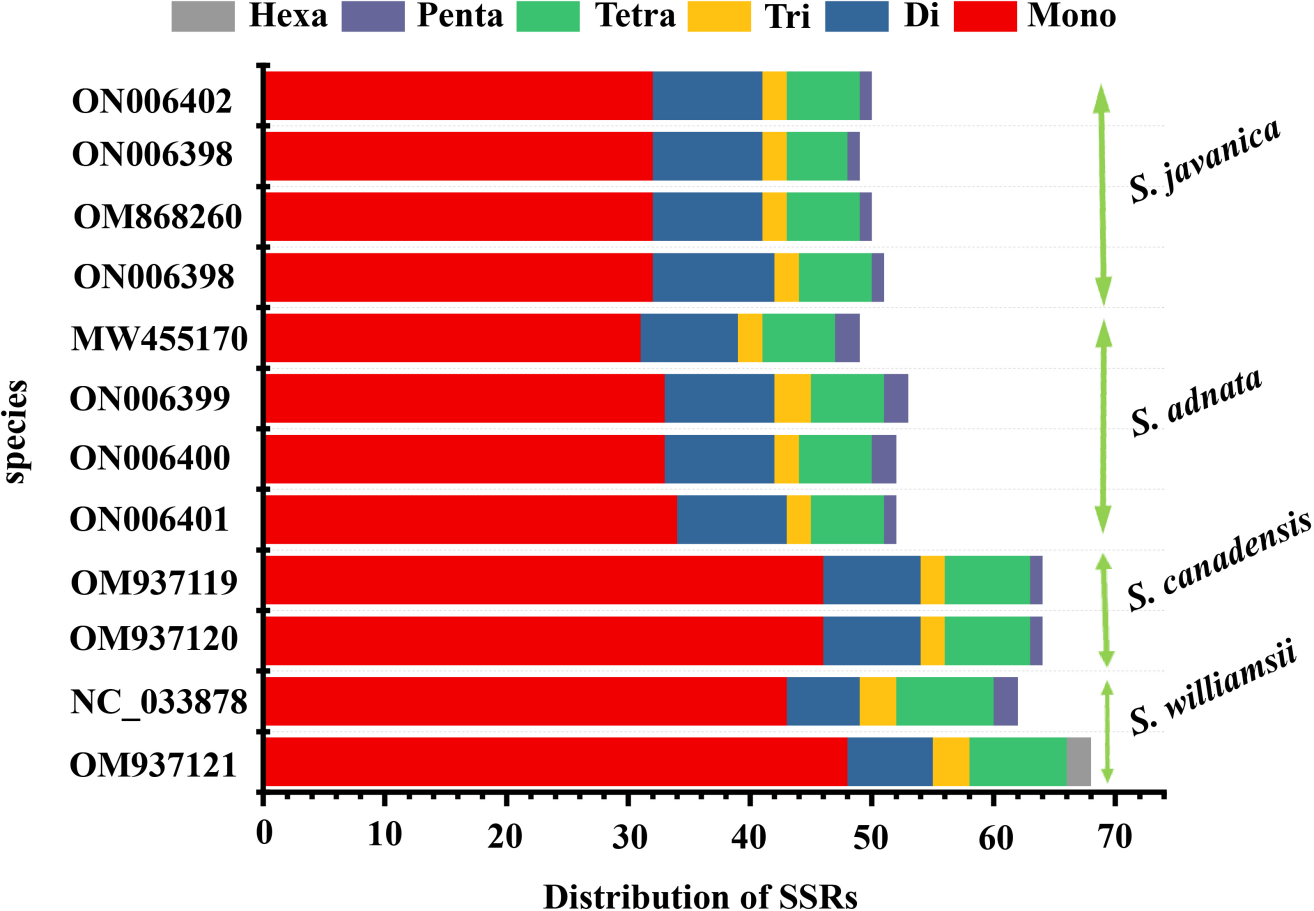
Analysis of SSRs in *Sambucus* chloroplast genomes. SSRs, simple sequence repeats.

In the repeat structure analysis, four types of long repeats including palindromic, forward, complementary, and reverse elements were identified in the studied *Sambucus* genomes **(** **Figure 4** **)**. Moreover, analysis of the microsatellites showed the presence of mononucleotide, dinucleotide, trinucleotide, tetranucleotide, and hexanucleotide SSRs in *Sambucus* cp genomes **(** **Table S3** **)**. *S. williamsii* (OM937121) had the highest number of repeats [68 SSRs], followed by *S. canadensis* [64 (OM937119 and OM937120) SSRs] and *S. adnata* [53 (ON006399) and 52 (ON006400 and ON006401) SSRs], while 49 (ON006398), 50 (OM868260), and 51 (ON006397) were found in *S. javanica*. Additionally, the variety of *S. javanica* and *S. chinensis* var. *pinnatilobatus* had a total of 50 SSRs. The most abundant nucleotide contents in all studied species were mononucleotide repeats (SSR loci A/T), followed by dinucleotides, tetranucleotides, trinucleotides, and pentanucleotides, whereas hexanucleotides exhibited the least number of SSRs. *S. williamsii* encompassed 48 mononucleotide, seven dinucleotide, three trinucleotide, eight tetranucleotide, and nine hexanucleotide repeats. Additionally, pentanucleotide repeats were absent in *S. williamsii* but were detected in the rest of the studied *Sambucus* species. Hexanucleotide repeats were present in *S. williamsii* but absent in other species **(** **Figure 5** **)**. The distribution pattern of SSRs between *S. adnata* and *S. javanica* exhibited high resemblances, and the hexanucleotide repeats were absent in both species **(** **Figure 5**; **Table S3**).

### 3.3 Expansion and contraction of inverted repeat regions

**Figure 6:**
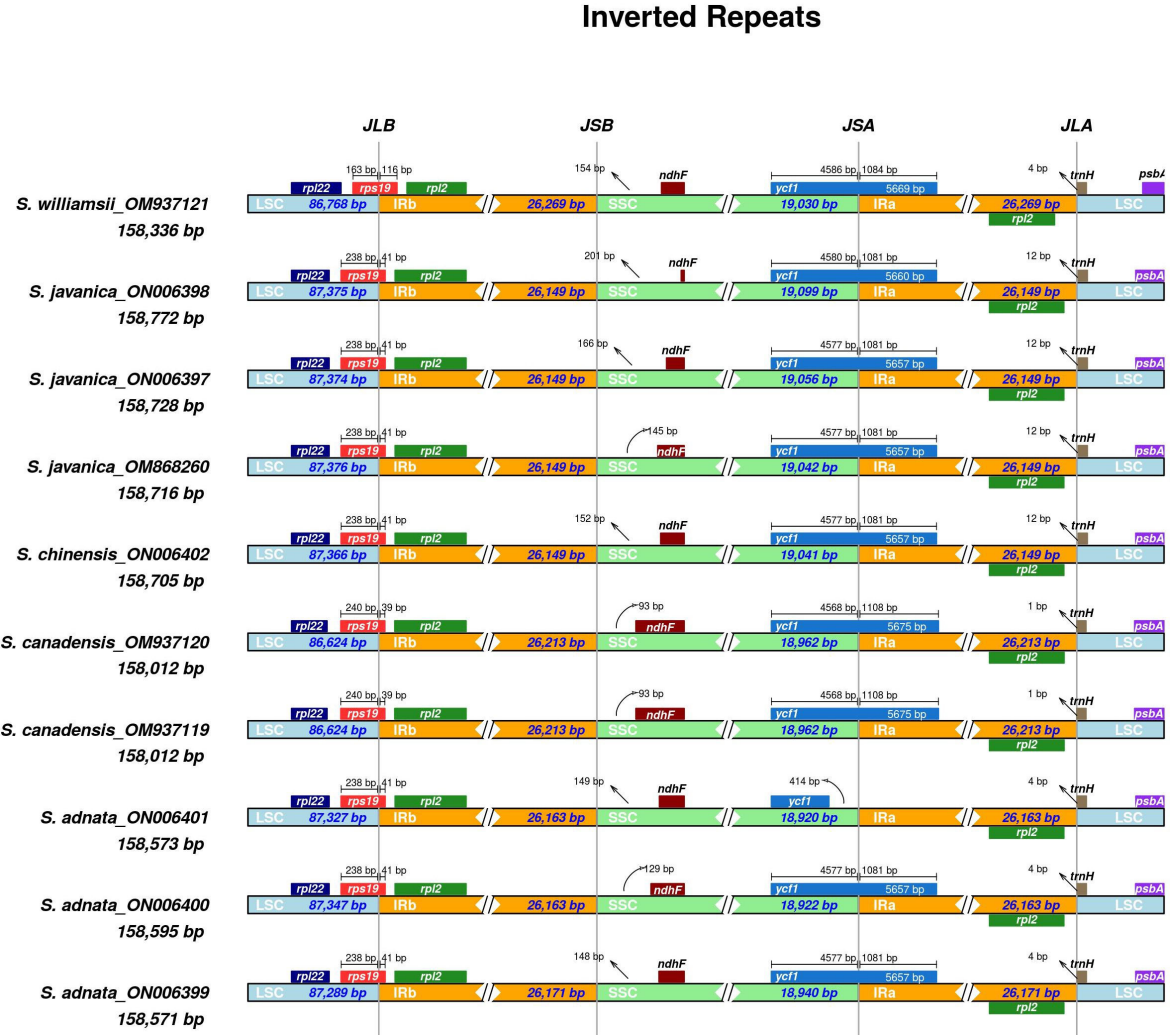
Comparison of the borders of LSC, SSC, and IR regions among *Sambucus* plastomes. LSC, large single copy; SSC, small single copy; IR, inverted repeat.

**Figure 7:**
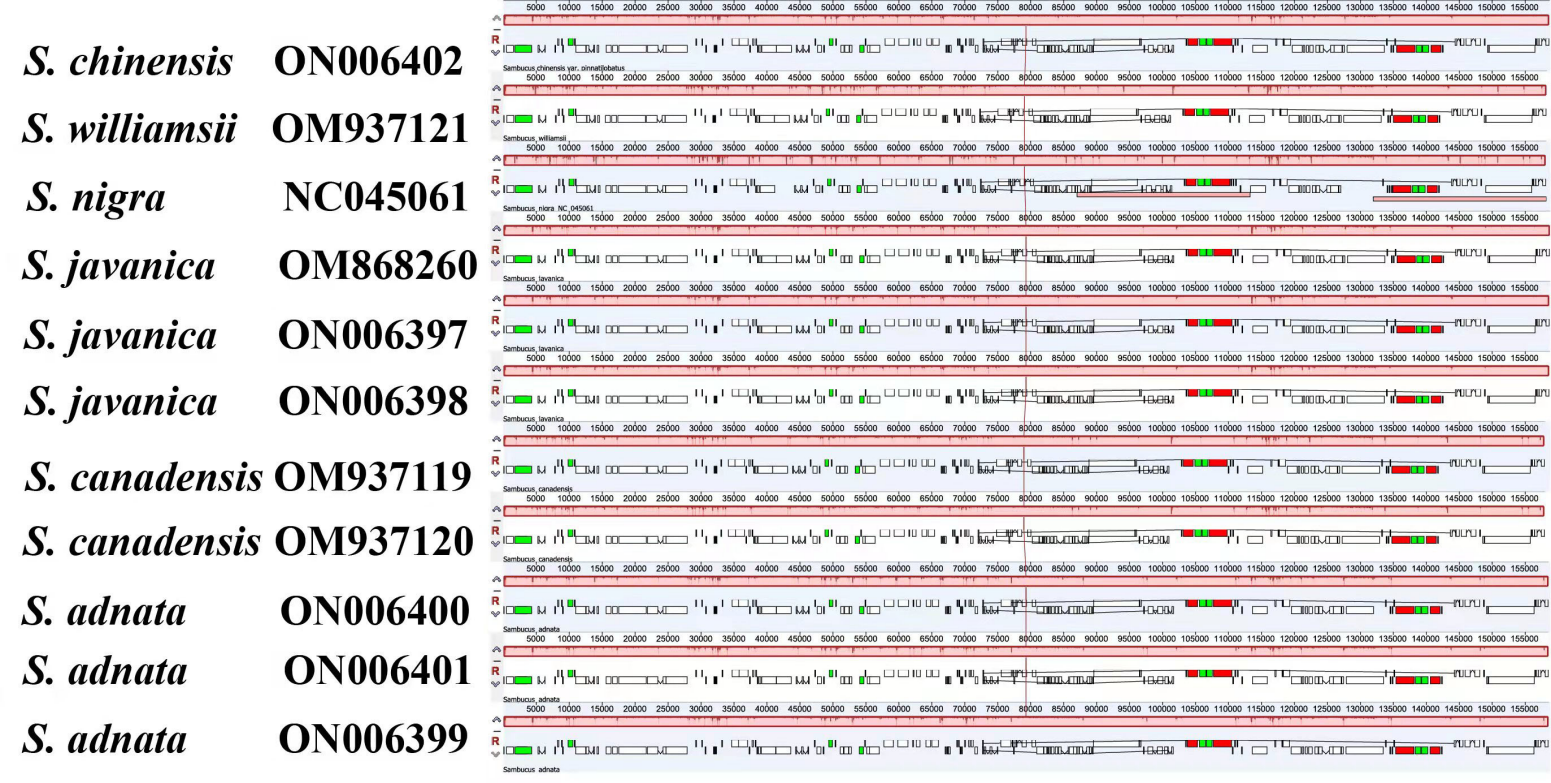
Structural genome comparison of the *Sambucus* species using Mauve program. The aligned DNA sequences positioned above the line are indicated in the clockwise direction while counterclockwise is presented below the line.

We performed comparisons between IR border regions of the four closely related *Sambucus* plastomes and six congeners. The detailed IR shift comparison was executed at the LSC/IRa, LSC/IRb, SSC/IRa, and SSC/IRb junction sites and the adjacent genes. Expansion/contraction of IRs was examined using *S. chinensis* (syn. *S. javanica*)_MW455170 as a reference genome. Our findings showed that LSC, IR, and SSC areas are slightly variable among *Sambucus* genomes **(** **Figures 6**, **7** **)**. Moreover, the sizes of *S. javanica* IR regions were identical (26, 149 bp), congruent to that of *S. chinensis* var. *pinnatilobatus*, a variety of *S. chinensis* (*S. javanica*). Furthermore, the populations of *S. canadensis* and *S. adnata* exhibited IR sizes of 26,213 and 26,163 bp, respectively, except for *S. adnata*_ON006399 (26,171 bp). *ndhF* gene was entirely situated within SSC and partially expanded in the range of 201 bp in *S. javanica*_ON006398 to 93 bp in *S. canadensis* (OM937120). The junction SSC/IRA (JSA) largely lies within *ycf1* gene that ranges from 4,586 bp in *S. williamsii*_MN937121 to 4,568 bp in *S. canadensis*_OM937120, whereas in *S. adnata*_ON006401, *ycf1* gene is located far from 414 bp in the SSC. *ycf1* pseudogene and *rps19* gene were present in all *Sambucus* species. In addition, *trnH-GUG* gene at the LSCs was far from the border JLA in the range of 1–12 bp **(** **Figure 6** **)**. *rpl2* gene was correspondingly positioned around IRb/LSC border, whereas *rps19* was located at the LSC/IRb junction site. The LSC/IRB (JSB) junction located within *rps19* gene largely lies in the range of 166 bp in *S. williamsii* _MN937121 to 39 bp in *S. canadensis*_MN937120.

### 3.4 Comparative analysis of chloroplast genomes

**Figure 8:**
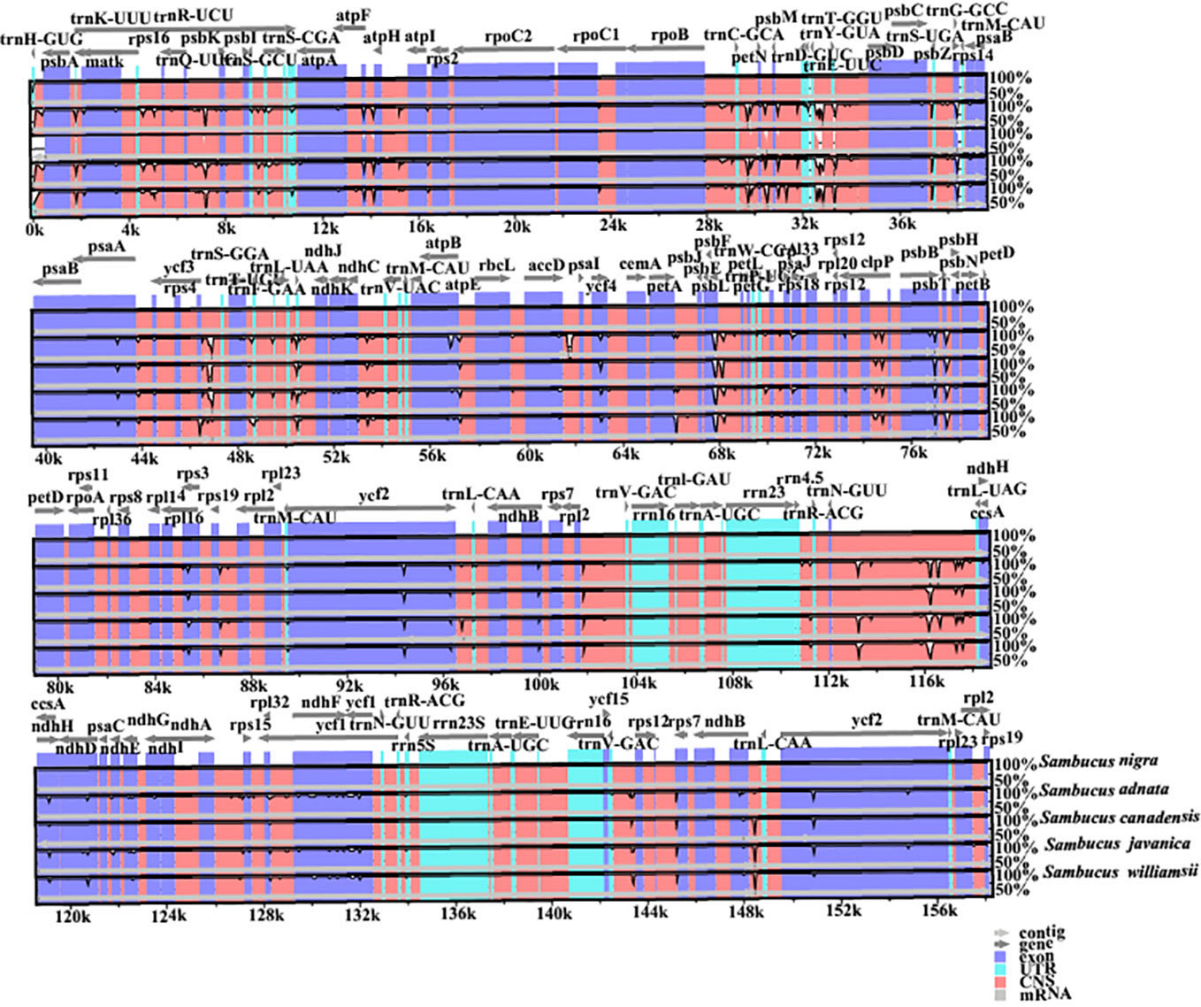
Visualized alignments of the four *Sambucus* species cp genomes. The sequence identity plot was made using mVISTA, with *Sambucus nigra* as a reference genome. The y-axis represents identity ranging from 50% to 100%.

Disparities in cp genome divergence between the four *Sambucus* species (*S. adnata* (ON006400), *S. canadensis* (OM937119), *S. javanica* (OM868260), and *S. williamsii* (OM937121)) were analyzed using the mVISTA program with the annotated sequence of *S. nigra*_NC_045061 as a reference genome **(** **Figure 8** **)**. The overall sequence identity of the plastomes indicated high consistency in the arrangement of genes, and the single-copy regions were more diversified and variable compared to the IR regions. The results showed high conservation in gene number, orientation, and order. Despite slight variations at the IR/SSC border, the conservative nature of IRs was evident. rRNA genes *rrn16*, *rrn23*, *rrn4.5*, and *rrn5* located at IRa and IRb were highly conserved. With the minority showing long repeats of more than 60 bp, most plastomes exhibited repeat lengths between 20 and 58 bp. The intergenic regions were more divergent, especially *atpH-atpA*, *petN-psbM*, *psbL-petG*, *trnV-rps12*, *rps16-psbK*, and *trn-GUU-ccsA* regions. Furthermore, the protein-coding regions including *ndhD*, *ndhH*, *ndhF*, *ycf4*, *psbB*, *psbL*, *psbP*, *rps3*, *ycf1*, and *ycf2* were highly divergent.

**Figure 9:**
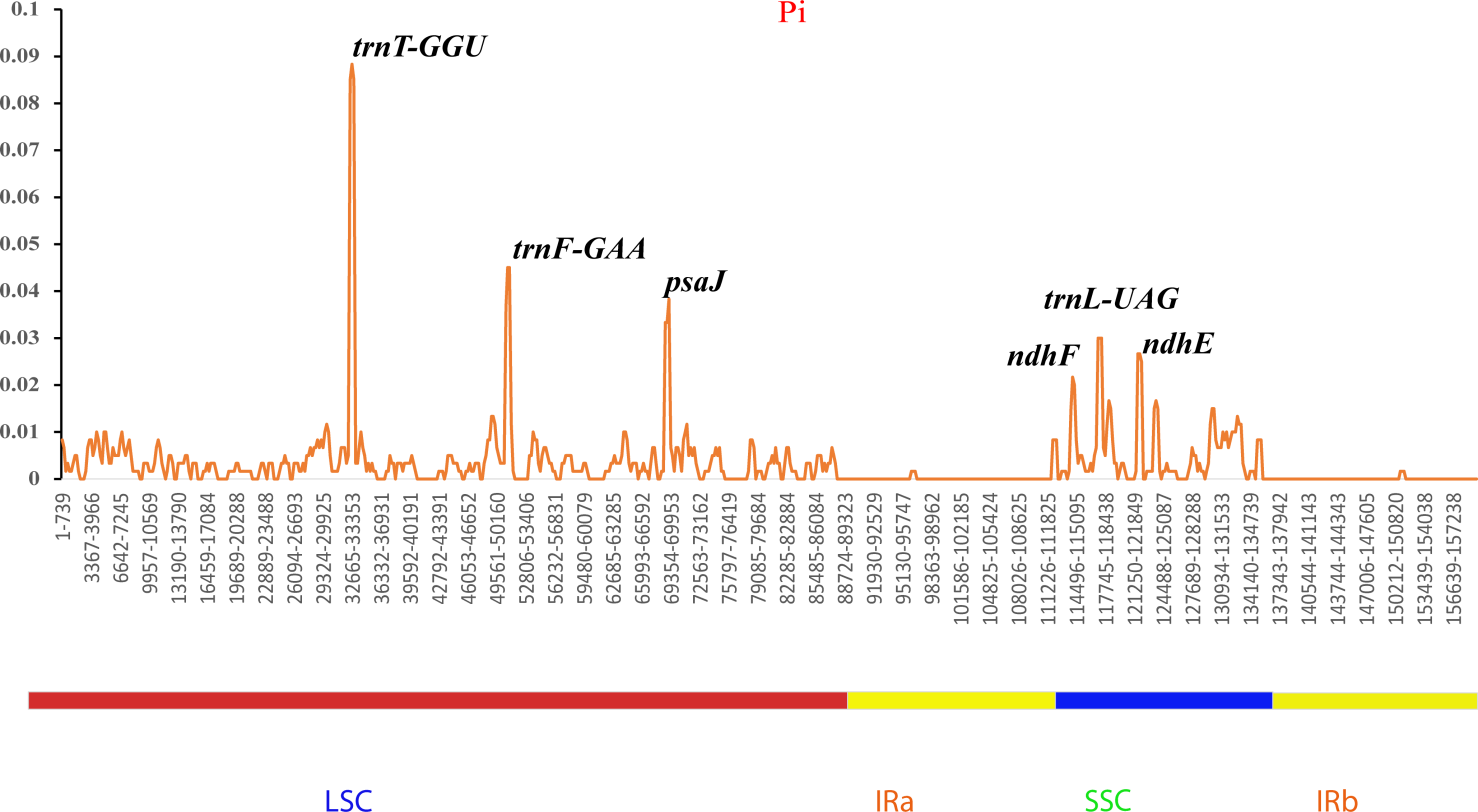
Sliding window analyses of the complete chloroplast genomes of five *Sambucus* species. The x-axis represents the position of the window, while the y-axis represents the nucleotide diversity (Pi) of each window (window length, 600 bp; step size, 200 bp).

Additionally, the nucleotide diversities of the cp genomes of five *Sambucus* species were compared using DNASP (Librado and Rozas, 2009), and the sequence variability (Pi) values were plotted using a reference annotation of *S. chinensis* (MW455170) **(** **Figure 9** **)**. The most divergent hotspots regions were *trnT-GGU*, *trnF-GAA*, *psaJ*, *ndhF*, *trnL-UAG*, and *ndhE* genes, situated in the coding and non-coding areas with the values of Pi values between 0 and 0.09. The LSC region exhibited higher divergence at *trnT-GGU* and *trnF-GAA* regions and *psaJ* genes (>0.04) compared to SSC (*ndhF*, *trnL-UAG*, and *ndhE*) regions. The SC regions displayed more variations than IR regions **(** **Figure 9** **)**. The nucleotide comparison between *S. javanica* and *S. chinensis* revealed negligible nucleotide variance (Pi = 0.00018) **(** **Table S4** **)**.

**Figure 10:**
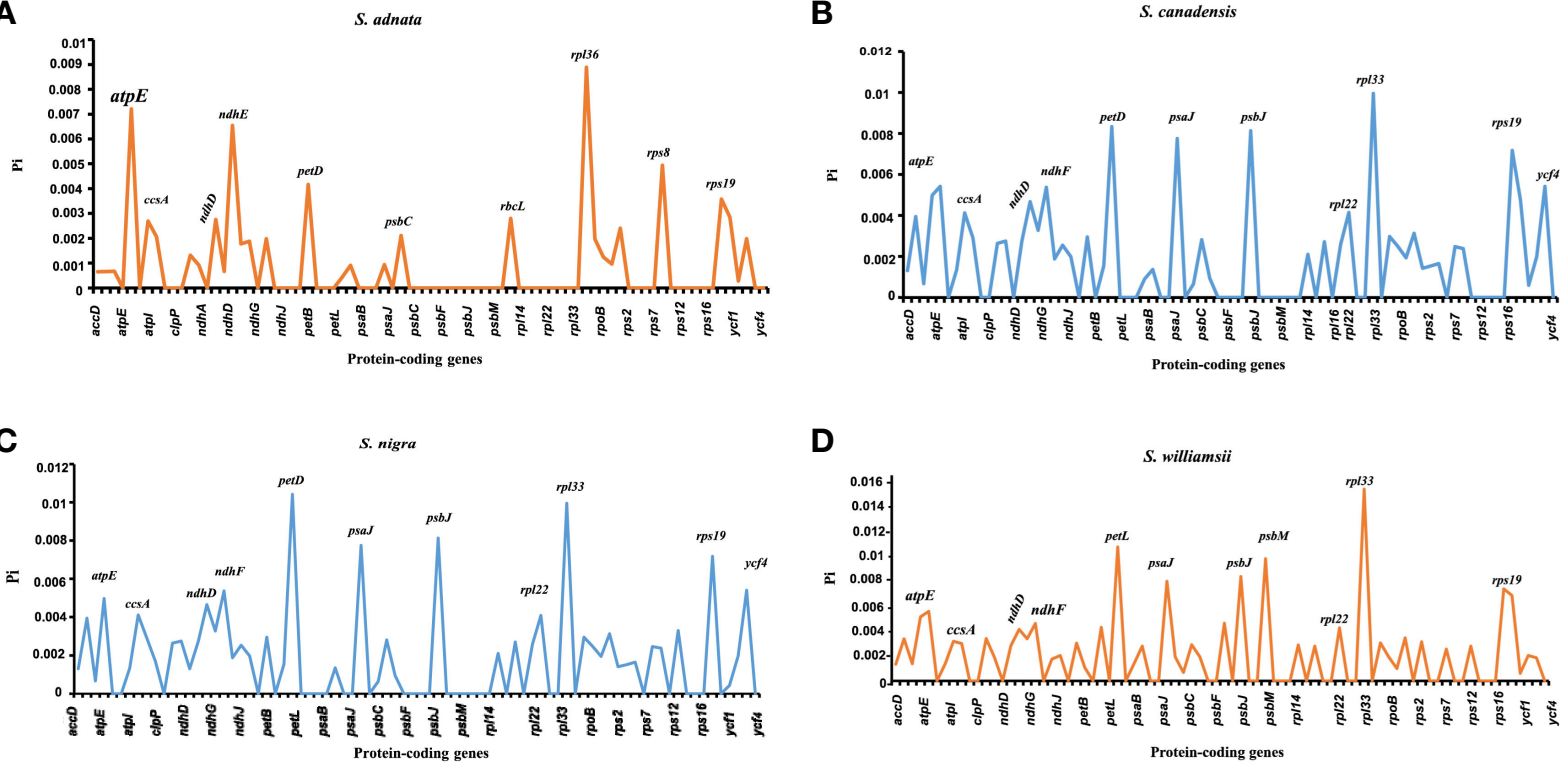
Nucleotide variability of the protein-coding genes of *Sambucus* plastomes. **(A)** The most divergent regions in *Sambucus adnata*. **(B)** The most divergent regions in *Sambucus canadensis*. **(C)** The most divergent regions in *Sambucus nigra*. **(D)** The most divergent regions in *Sambucus williamsii*.

The nucleotide diversity of protein-coding genes extracted from five *Sambucus* species indicated several genes including *atpE*, *ccsA*, *ndhD*, *ndhF*, *petD*, *psaJ*, *psbJ*, and *rpl33* as highly variable **(** **Figure 10** **)**. Conversely, *S. javanica_*OM868260 and *S. chinensis_*MW455170 (reference genome) exhibited zero nucleotide variability (Pi = 0).

### 3.5 Codon usage analysis

**Figure 11:**
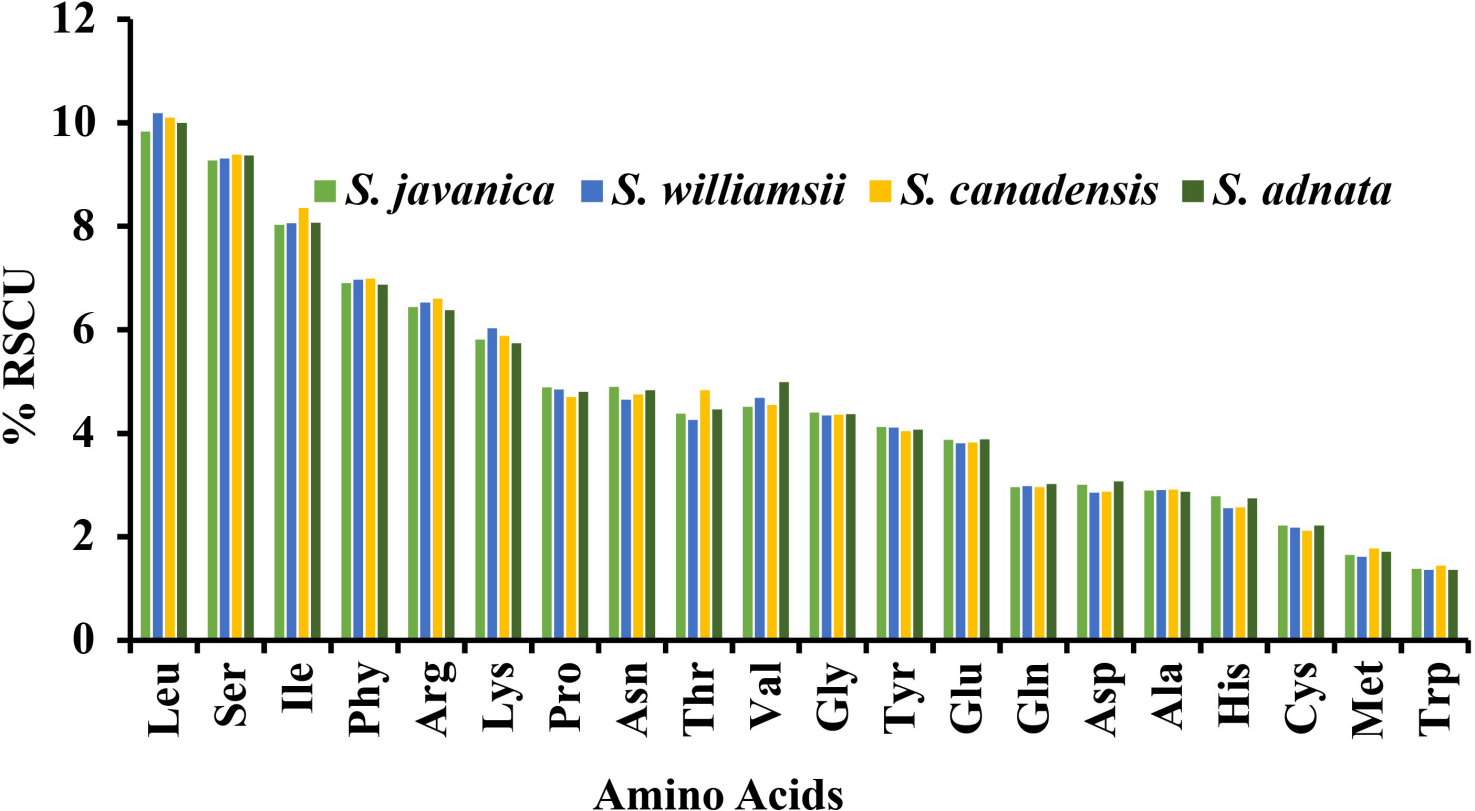
Percentage RSCU of amino acids in *Sambucus* species.

A comparison was made between selected populations of *Sambucus* species including *S. javanica* (OM868260), *S. williamsii* (OM937121), *S. canadensis* (OM937119), and *S. adnata* (ON006399). The number of codons ranged from 52,670 to 52,909, and both species exhibited 64 different types of codons **(** **Table S5** **)**. We found 20 different amino acids (AAs) in all studied species **(** **Figure 11** **)**, which revealed high similarities in the distribution patterns. Leucine, encoded by six codons, was the most abundant AA and attributed to an average of 9.83%–10.19%. Encoded UUA exhibited the highest Relative Synonymous Codon Usage (RSCU) values, and the ATG codon was found to highly occur as a start codon. The absence of bias was exhibited in methionine and tryptophan, which were encoded for ATG and UGG, respectively (RSCU = 1.00). In addition, the codon bias of most AAs had high preferences (RSCU > 1). Moreover, higher encoding of serine was also exhibited, while the rarest encoding was revealed in cysteine. The RSCU between *S. javanica* and *S. adnata* showed high resemblances in the encoding pattern.

### 3.6 Adaptive evolution analysis

**Figure 12:**
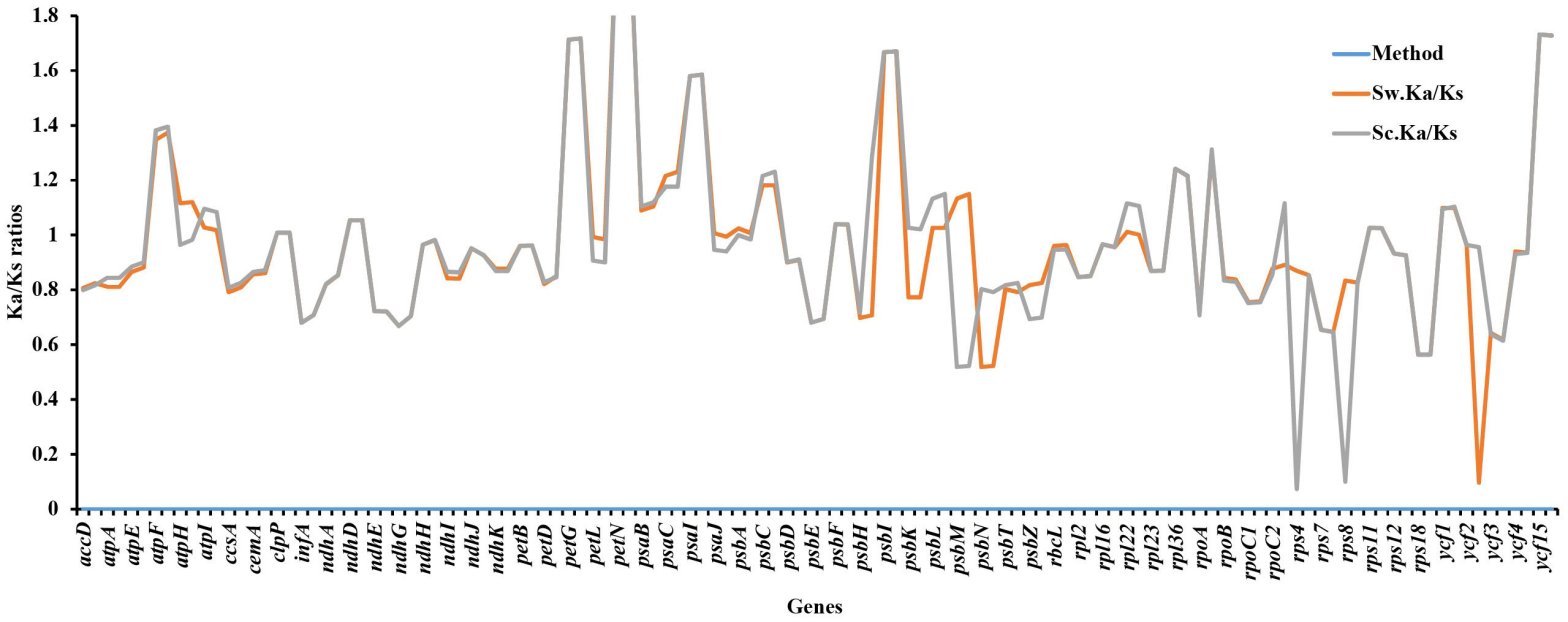
Synonymous and non-synonymous substitution rates of *Sambucus*. Note: Sc represents *Sambucus chinensis*, while Sw denotes *Sambucus williamsii.*.

**Figure 13:**
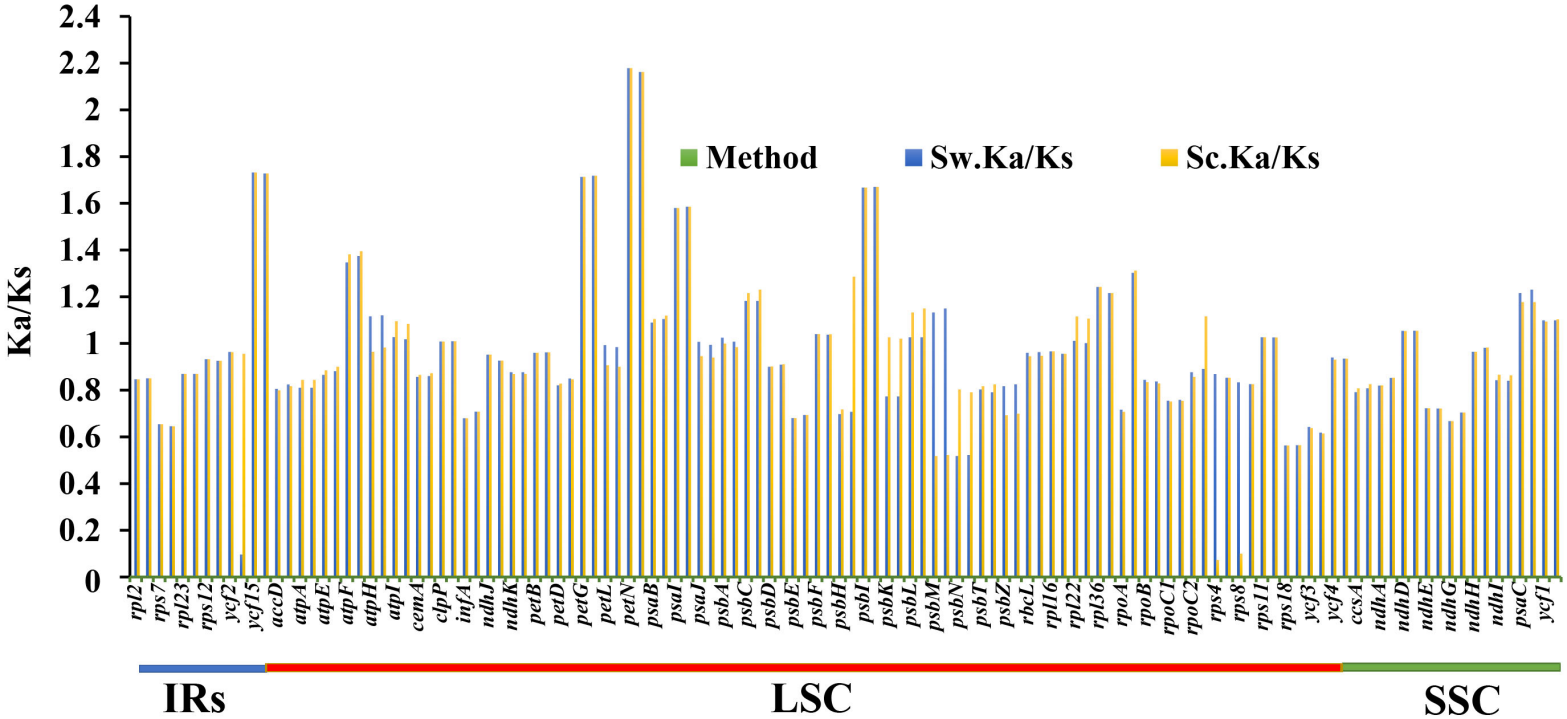
Ka/Ks substitution ratios in the LSC, SSC, and IRs. Sc represents *Sambucus chinensis*, while Sw denotes *Sambucus williamsii.* LSC, large single copy; SSC, small single copy; IRs, inverted repeats.

Ka/Ks ratios indicate selection pressures and are significant in determining whether a mutation is beneficial, purifying, or neutral. Eighty-one protein-coding genes were extracted from *S. williamsii* (OM937121), and the values of Ka and Ks were evaluated using *S. chinensis* (MW455170) as a reference genome. Eighteen photosynthetic genes (*atpB*, *matK*, *ndhB*, *ndhC*, *ndhF*, *petA*, *psaA*, *psbB*, *psbZ*, *rpl14*, *rpl20*, *rpl32*, *rpl33*, *rps2*, *rps3*, *rps14*, *rps15*, *rps16*, and *rps19*) exhibited zero Ka, Ks, and Ks/Ks substitutions (Ka = 0, Ks = 0, and Ka/Ks = 0). The Ka and Ks values and the substitution rates (Ka/Ks) of the remaining 60 protein-coding genes were calculated **(** **Figure 12**; **Table S6** **)**. The findings showed that a higher average was recorded in Ks (1.068651) than in Ka (0.985531). Twenty-one positively selected genes encompassing *atpF*, *atpH*, *atpI*, *clpP*, *ndhD*, *petG*, *petN*, *psaB*, *psaC*, *psaI*, *psbA*, *psbC*, *psbF*, *psbI*, *psbK*, *psbL*, *rps11*, *rpl22*, *rpoB*, *ycf1*, and *ycf15* were obtained. Averagely, low rates of Ka/Ks substitutions were displayed by most genes (0.970625), and the high Ka/Ks substitution rates were observed in the LSC and SSC regions compared to the IRs **(** **Figure 13** **)**.

### 3.7 Phylogenetic analysis

**Figure 14:**
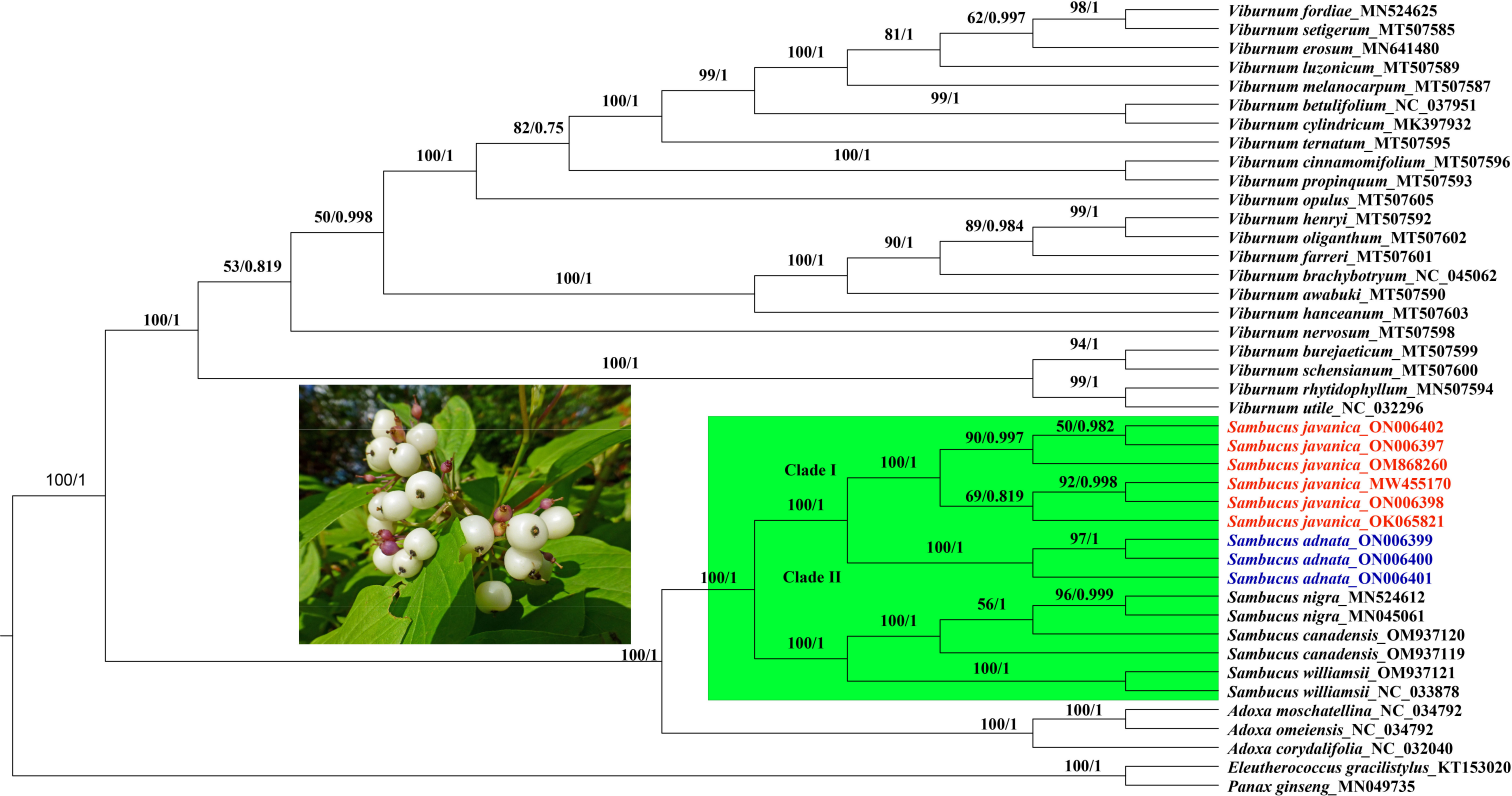
Phylogenetic tree reconstruction based on 58 shared protein-coding genes of 42 Viburnaceae species. The numbers indicate bootstrap values from the ML (left) and BI (right). *Eleutherococcus gracilistylus* and *Panax ginseng* were used as outgroups. ML, maximum likelihood; BI, Bayesian inference.

In the present study, the whole plastomes as well as 58 shared protein-coding genes extracted from complete cp genomes of 40 Viburnaceae species and two outgroups from Araliaceae were selected to reconstruct phylogenetic trees. Similar tree topologies were yielded in all datasets by the ML and BI methods and confirmed the monophyly of *Sambucus* **(** **Figures 14**; **S2** **)**. The phylogenetic relationship within the family recovered two major groups encompassing the *Viburnum* (larger group) and *Sambucus*–*Adoxa*–*Tetradoxa*–*Sinadoxa* genera (Adoxoideae) (smaller group) and was significantly supported with bootstrap values greater than 78/100 **(** **Figure 14** **)**. In the Adoxoideae group, *Adoxa*–*Tetradoxa*–*Sinadoxa* (Adoxina) was closely related to *Sambucus.* The taxa *S. williamsii*, *S. nigra*, *S. adnata*, and *S. javanica* clustered into distinct clades. In this study, the population encompassing *S. javanica* was treated as clade I, while clade II included the population of *S. adnata*. Individuals of *S. chinensis* and *S. javanica* were clustered together in the same clade in ML, BI, and NN analyses; thus, *S. chinensis* was treated as a conspecific/synonym to *S. javanica*. The population of *S. nigra* clustered together as monophyletic with inconsistent nesting of one individual of *S. canadensis* (OM937119) in the same clade, while the other (*S. canadensis*_OM937120) separated from the latter in both datasets. Therefore, *S. canadensis* might be closely related to *S. nigra.* Moreover, *S. williamsii* clustered into a separate monophyletic clade.

**Figure 15:**
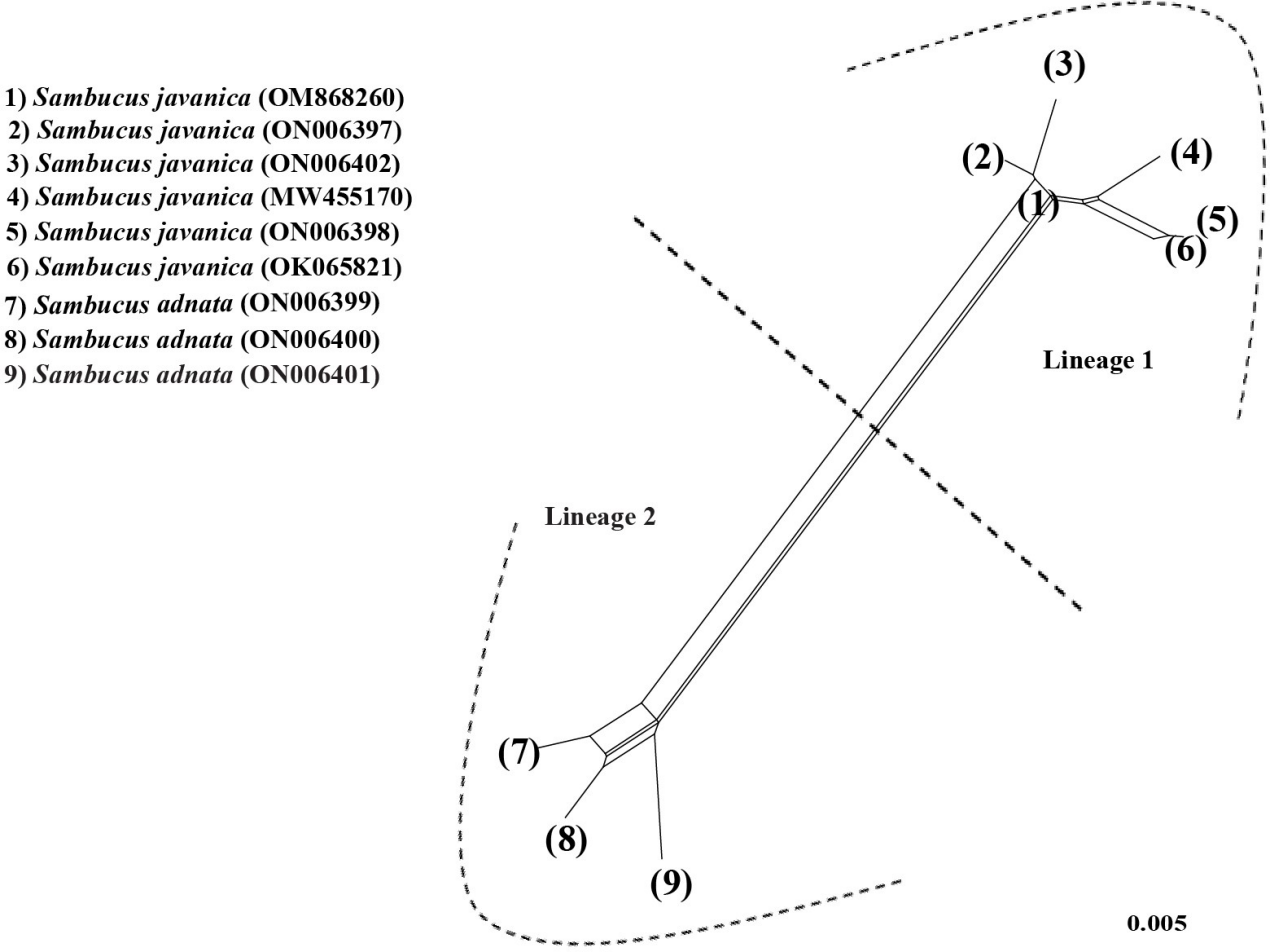
Neighbor-Net for protein-coding sequences of *Sambucus*.

NN analysis was used to determine the phylogenetic resolution between the two closely related *S. adnata* and *S. javanica*. In the NN diagram, the term “lineage” was used to denote clustered populations. Individuals of *S. javanica* (1, 2, 3, 4, 5, and 6) (trivially lineage 1) were distinct and remained isolated from the rest of the population (7, 8, and 9) encompassing *S. adnata* (lineage 1) **(** **Figure 15** **)**. The CDS split graph showed two major groups, and lineage 1 corresponds to clade I, while the populations of *S. javanica* (lineage 1) correlate to clade II in **Figures 9**; **S1**. Nonetheless, the population comprising lineage 1 and clade I appears in close proximity to each other, exhibiting a great genetic affinity.

## 4 Discussion

### 4.1 Organization and comparison of genomic features

In the present study, we sequenced the cp genomes of four *Sambucus* species, annotated and assembled the cp genomes, identified the SSRs within the genomes, analyzed the codon usage and adaptive evolution, and performed comparative phylogenetic analysis within the family Viburnaceae. The findings showed that all the cp genomes presented a typical quadripartite conformation congruent to vast angiosperms (Park et al., 2019; Ran et al., 2020). They were highly conserved in terms of structure, organization, and gene content, with only a few differences in their sizes primarily caused by evolutionary events (IR contraction and expansion) at the border regions (Shaw et al., 2014; Rabah et al., 2019; Abdullah et al., 2020).

The inclusive GC of 38% was detected in the cp genomes of all *Sambucus* species and collaborated with the previously reported values in the family Viburnaceae (Fan et al., 2018). Additionally, IR regions exhibited higher estimated GC contents when compared to the LSC and SSC regions. As previously reported, the high content of GC nucleotides in several genes in IR regions could be possibly linked to the higher GC percentages (Curci et al., 2015; Raman et al., 2017; Fu et al., 2022).

Based on the gene contents, 132 genes were found in all *Sambucus* cp genomes, encompassing 37 tRNA, eight rRNA, and 87 CDs, and their arrangements were largely consistent with the majority of the previously reported cp genomes in the genus (Fan et al., 2018; Ran et al., 2020). The evolution of plant lineages is a dynamic process in IR contraction/expansion at the boundaries of the cp genomes and plays a vital role in revealing evolutionary events (Oyebanji et al., 2020; Bai et al., 2021). In our findings, it was evident that IR contraction/expansion varies at the boundaries, and this character is a synapomorphy of the 10 studied plastomes, consistent with several angiosperms (Shaw et al., 2014; Amenu et al., 2022). *Sambucus* genomes reported no gene loss, and the presence of *ycf1* pseudogene might be due to evolutionary events at the IRs (Ran et al., 2020). Interestingly, *rps12* gene had a unique trans-splicing with LSC incorporating the 5′ end of the exon and 3′ end positioned in the IRs, a phenomenon highly detected in most plants (Hildebrand et al., 1988; Munyao et al., 2020). *infA* gene usually participates in the initiation of the translation process (Gichira et al., 2017; Chen et al., 2019). In the present study, this gene was found to be non-functional, congruent to the previous related investigations on other plants (Ewels et al., 2016; Shen et al., 2016; Amiryousefi et al., 2018). Nonetheless, it is suggested to be translocated into the nuclear plastome genome in an active form that is functional or sometimes its copy (Piot et al., 2018; Shahzadi et al., 2019).

### 4.2 Analysis of repeats and codon usage

Dispersed repeat sequences are essential in the rearrangement of genomes and thus form a basis for resolving the phylogenetic complexities among different taxa (Raman et al., 2017). They are normally used as commendable molecular markers in plant taxonomy (Gu et al., 2019). Our present study assessed the dispersion of repetitive sequences in several *Sambucus* cp genomes, which showed similarities in SSR motif distribution, congruent to previous findings (Abdullah et al., 2020; Mehmood et al., 2020a). Palindromic repeats were the richest among studied species, similar to previous outcomes in the *Sambucus* genus (Fan et al., 2018; Ran et al., 2020). Oligonucleotide repeats (SSRs) usually generate mutations in genomes and thus are regarded as proxies to identify mutational hotspots (Ahmed et al., 2012; Abdullah et al., 2020; Mehmood et al., 2020b). Our results indicated that most SSRs are positioned at the non-coding spacers (NCSs) and thus could be potential DNA markers for species identification (Fan et al., 2018; Ran et al., 2020).

Codon usage refers to how an organism encodes amino acids in its protein genes using identical codons (Munyao et al., 2020). However, several previous studies showed variabilities in codon usage among different species (Li et al., 2016; Srivastava and Shanker, 2016). Codons link proteins and nucleic acids, thus acting as significant transmitters of genetic information used in plastome evolution (Liu et al., 2005). Each gene in an organism has its favorite amino acid codon, which is referred to as codon use bias (Wu et al., 2007), which is greatly influenced by natural selection (Liu and Xue, 2005). Our findings revealed high RSCU values for the codons with A/T at the 3′ end in place of C/G, similar to previous observations in several cp genomes of land plants (Abdullah et al., 2020; Mehmood et al., 2020a), and it is presumably influenced by enormous AT content in the plastid genomes. The present study showed high proportions of leucine and isoleucine, while cysteine exhibited the least AA. The cp genomes of *Sambucus* species established similarities in codon usage, and the codons ending in A/U had higher encoding efficacy than those ending in C/G. Previous studies indicated that close phylogenetic relationships among closely related species may be due to similarities in codon selection strategies (Qian et al., 2013). Cui et al., 2019; Fu et al., 2022 In this study, the codon bias of most AAs was highly preferred (RSCU ≥ 1), suggesting that codon usage happens more often than expected. RSCU ≥ 1 was notably displayed by the majority of codons ending with A/U. The codon usage is less often than expected when values of less than one (RSCU < 1) are exhibited (Li et al., 2017). RSCU < 1 was exhibited for most codons ending with C/G in this study. Moreover, the codon usage of methionine and tryptophan was not preferred (RSCU = 1).

### 4.3 Comparative genome characterization analysis

The various regions of the chloroplast DNAs (cpDNAs) were reported as possible markers to explore the phylogenetic relationships of the closely related species (Gu et al., 2019). Some of the hypervariable regions in the cpDNAs including *ycf1*, *trnF-ndhJ*, *rpl33*, *rps2-rpoC2*, *rps18-rpl20*, *rps16*, *trnG-trnR*, *atpE*, *trnM-psbD*, *ccsA*, *trnN-ndhF*, *clpP*, *ycf4-cemA*, *ycf4*, *ndhG-ndhI*, *ndhF*, *rpl32trnL*, *ndhD*, *atpI-rps2*, and *rpl16* were stated as significant markers in studying the phylogenetic relationships of *Sambucus* species (Fan et al., 2018; Ran et al., 2020). In the present study, the most divergent regions were *trnT-GGU*, *trnF-GAA*, and *trnL-UAG* intergenic spacers as well as *atpE*, *ccsA*, *ndhD*, *ndhF*, *petD*, *psaJ*, *psbJ*, and *rpl33* genes. The proportion of variable sites in non-coding regions was higher than in the coding regions, consistent with the previous findings by Fu et al. (2022). Hence, they can be employed as candidate DNA barcodes for phylogenetic and phylogeographic studies. The SC regions were more variable than IR regions and thus less conserved. In this study, the most informative plastome region was *trnT-GGU* and thus could be a useful marker in phylogenetic resolution at lower-level phylogenetic studies.

The protein-coding genes have been widely used to determine selection pressures (Wang et al., 2021). Under positive selection in the Ka/Ks analysis, 21 identified genes were correlated with adaptive evolution. High evolution rates were observed in functional genes with high Ka/Ks ratios, whereas the genes that were linked to photosynthesis exhibited slow evolutionary rates, agreeing with the results obtained by Song et al. (2022). The latter exhibited no obvious nucleotide changes (Ka and Ks = 0), indicating that *Sambucus* plastomes were relatively conserved. Most genes exhibited Ka/Ks values of less than one, suggesting that they may have experienced extensive purifying selection and could remove deleterious mutations (Wu et al., 2020).

### 4.4 Phylogenetic analysis

Phylogenetic relationships within Viburnaceae were established and resolved by several authors in the past few decades (Chase et al., 1993; Eriksson and Donoghue, 1997; Donoghue et al., 2001; Jacobs et al., 2010). The complete cp genomes and protein-coding genes dataset have been previously utilized to reconstruct phylogenies (Blazier et al., 2016; Hou et al., 2016; Namgung et al., 2021). In this study, we used complete cp genomes and protein-coding genes to examine the phylogenetic relationships between several *Sambucus* species by BI, ML, and NN methods.

Our findings revealed significantly supported clades within Viburnaceae and confirmed the monophyly of *Sambucus*, similar to previous findings (Fan et al., 2018; Ran et al., 2020). The obtained topologies based on different analyses exhibited similar branching patterns, consistent with prior studies within the family (Lens et al., 2016; Ran et al., 2020). In the present work, we adopted the most recent taxonomic treatments of *Adoxa*, *Tetradoxa*, and *Sinadoxa*. Based on this treatment, *Tetradoxa* L. and *Sinadoxa* L. are circumscribed within *Adoxa* L., which is regarded as an accepted genus encompassing four recognized species including *A. corydalifolia* (C.Y.Wu, Z.L.Wu & R.F.Huang) christenh. & Byng (*S. corydalifolia*), *A. moschatellina* L., *A. omeiensis* H. Hara (*T. omeiensis* (H. Hara) C.Y.Wu), and *Adoxa xizangensis* G.Yao (POWO, 2023). Therefore, *Tetradoxa* C.Y.Wu, and *Sinadoxa* C.Y.Wu, Z.L.Wu & R.F.Huang are treated as synonyms of *Adoxa* L.

Previous morphological and molecular studies showed a close relationship between *S. adnata* and *S. javanica*, which were clustered in the same clade (Eriksson and Donoghue, 1997; Lens et al., 2016). The findings by Ran et al. (2020) inferred by the complete chloroplast genome sequence dataset indicated that *S. javanica* Blume is a close relative of *S. adnata* Wall. ex DC. The latter are morphologically united by valvate corolla lobes, yellow anthers, bladelike stipules, umbellate cymes, three-lobed stigma, and urceolate calyx (Eriksson and Donoghue, 1997; Silalahi and Wakhidah, 2021); thus, difficulties may arise during the discrimination process. In this study, molecular analysis showed amplification of primers in *S. adnata* that were distinct from those amplified by *S. javanica* and segregated into different clades. The segregation between *S. javanica* and *S. adnata* is supported by several morphological characteristics such as inconspicuous stem lenticels, two to three pairs of leaflets, flower filaments connate at base, and fruits characterized by verrucate pyrenes, which distinguish *S. javanica* from *S. adnata* (http://www.efloras.org/).

Populations of the same species tend to cluster together within the tree, and possible reticulation events can be evidenced by the NN phylogenetic networks (Bryant and Moulton, 2002). The phylogenetic relationships indicated by edges revealed a complete divergence between the populations of *S. javanica* and *S. adnata*. Although *S. adnata* appears closely related to *S. javanica*, we treat them as distinct species due to existing morphological differences and their discrimination into different clusters and/or lineages observed in ML, BI, and NN analyses. The results obtained in both analyses indicate similarities in the retention of distinct gene pools between *S. javanica* and *S. adnata* and thus should be treated as discrete taxonomic entities. Moreover, negligible nucleotide variability between *S. javanica* and *S. chinensis* was observed in the current study. Thus, we confirm *S. chinensis* as a synonym for *S. javanica*, congruent to previous findings (Kern and van Steenis, 1948; Ohashi, 2015; WFO, 2023). Representatives of the *Sambucus* genus frequently show slight variations within individual species (Elkiran, 2021). Consequently, the infraspecific classification by Ohashi (2015) regarded *S. chinensis* as a form of *S. javanica* due to the sole distinctive morphological attribute of fruit color (black or blackish purple), distinguishing the latter from *S. javanica* (red). However, *S. chinensis* was considered a synonym of *S. javanica* subsp. *chinensis* (Lindl) Fukuoka and conspecific to *S. javanica* (POWO, 2023). The present findings support the conspecific nature of *S. chinensis* and *S. javanica*. Our results indicate the close relatedness of the medicinally significant *S. nigra* and *S. canadensis*, consistent with previous findings (Eriksson and Donoghue, 1997; Jacobs et al., 2010). Therefore, more specimens will be required to further analyze their taxonomic treatment in the future.

## 5 Conclusion

In the present study, the newly sequenced cp genomes of the populations of *S. javanica*, *S. adnata*, *S. canadensis*, and *S. williamsii* were reported and combined with 16 others to perform the comparative molecular analysis of 40 Viburnaceae cp genomes. A comparative phylogenetic analysis of the studied species was concluded. The structure, order, and gene content of the cp genomes of *Sambucus* species exhibited striking similarities, revealing their highly conservative nature. This study showed that *trnT-GGU*, *trnF-GAA*, *psaJ*, *ndhF*, *trnL-UAG*, and *ndhE* genes as the most divergent hotspot regions. The analyzed hypervariable regions, SSRs, and repeat sequences could be utilized as possible markers for molecular phylogeographic and genetic studies. Moreover, the phylogenetic analysis showed complete segregation of Viburnaceae species into two major branches representing *Viburnum*, *Sambucus*, and *Adoxa* (Adoxoideae) groups. *Adoxa* L. encompassed the *Adoxa*–*Tetradoxa*–*Sinadoxa* (Adoxina) group. Populations of different *Sambucus* species clustered separately, with the exception of *S. canadensis*, while individuals of the same species clustered clearly in the same clade. Further, the classical taxonomy of *Sambucus* based on protein-coding genes and whole cp genomes supported the separation of *S. javanica* Blume and *S. adnata* Wall. ex DC. Therefore, the results presented in this work demonstrate that the complete cp genomes and protein-coding genes could be used to discriminate species. The findings of this study provide rich genetic data for phylogenetic investigations, which might be useful for future research within Viburnaceae.

## Data availability statement

The data presented in this study are deposited in the Genbank repository. The accession number(s) can be found in the article/**Supplementary material**.

## Author contributions

EW, G-WH, and Q-FW participated in the study de sign and carried out experiments. EW and G-WH collected plant specimens. WO, SA, EMM, and ESM contributed to the drafting of the manuscript and data analysis. SM and S-XD revised the draft manuscript. All authors read and approved the final version of the manuscript.
