# Supplementary figures and images for: Comparative chloroplast genome analysis of Sambucus L. (Viburnaceae): inference for phylogenetic relationships among the closely related Sambucus adnata Wall. ex DC Sambucus javanica Blume
Source: Front Plant Sci. 2023 Jun 16;14:1179510. doi: 10.3389/fpls.2023.1179510 (PMC10313135; doi:10.3389/fpls.2023.1179510)

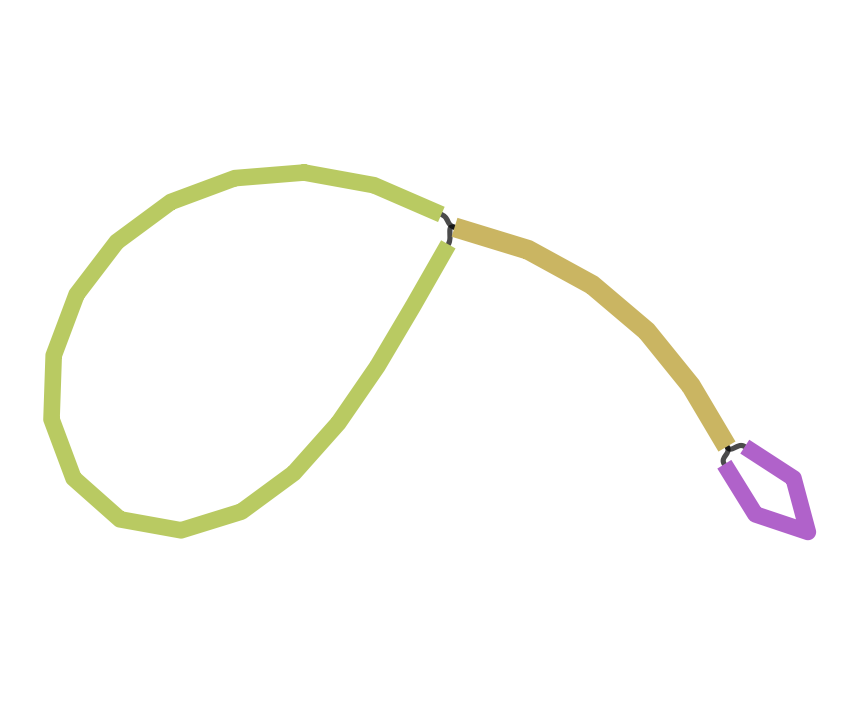

Supplement: Supplementary file 1 [file Image_1.png]

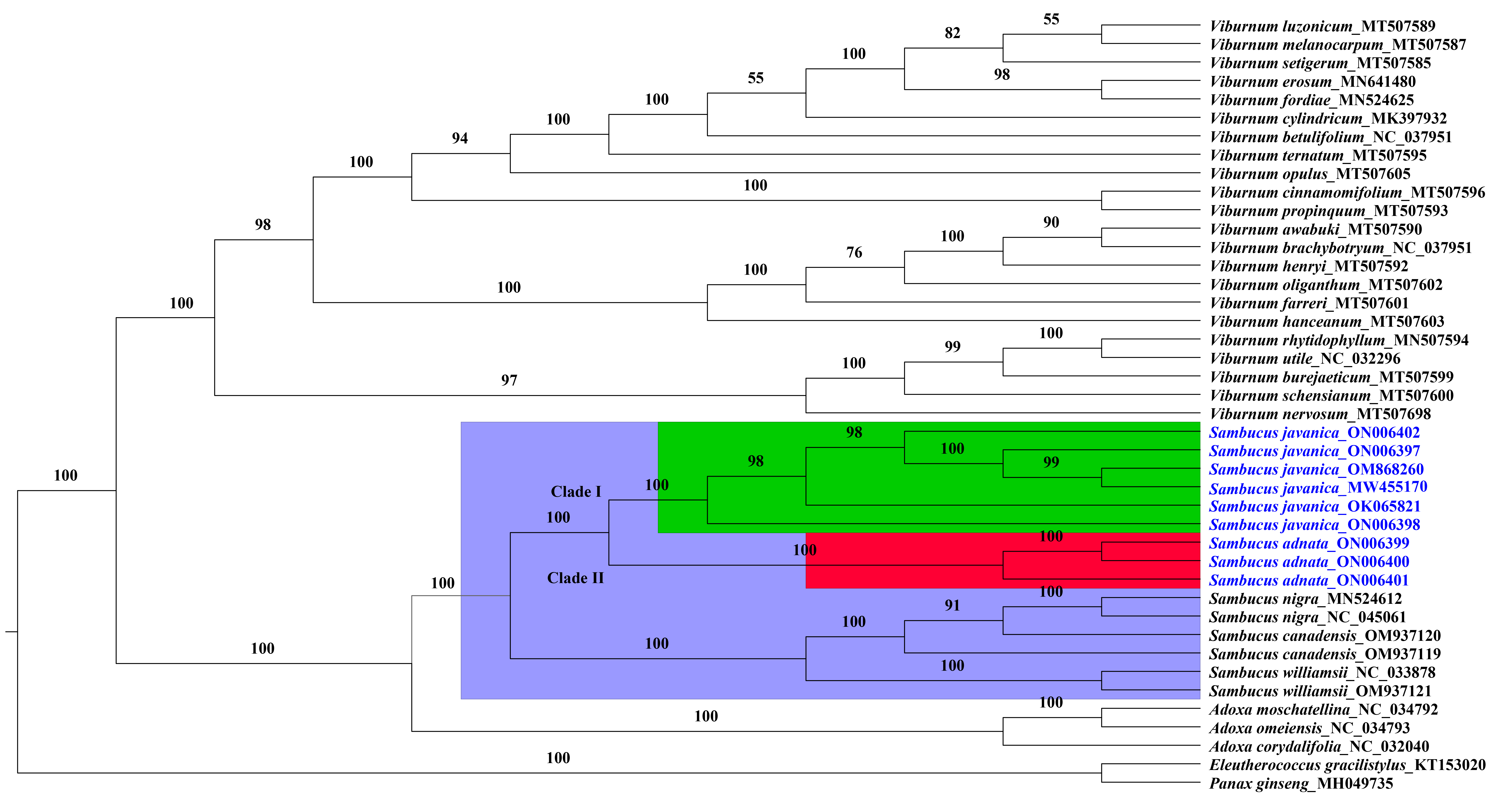

Supplement: Supplementary file 2 [file Image_2.jpeg]
