# Supplementary material for: Comparative chloroplast genome analysis of Sambucus L. (Viburnaceae): inference for phylogenetic relationships among the closely related Sambucus adnata Wall. ex DC Sambucus javanica Blume
Source: Front Plant Sci. 2023 Jun 16;14:1179510. doi: 10.3389/fpls.2023.1179510 (PMC10313135; doi:10.3389/fpls.2023.1179510)
Supplement: Supplementary file 3 [file Table_1.docx]

Supplementary Material

**File S1** The text generated following the assembly of the complete chloroplast genomes of *Sambucus*.

GetOrganelle v1.7.5.3

get_organelle_from_reads.py assembles organelle genomes from genome skimming data.

Find updates in https://github.com/Kinggerm/GetOrganelle and see README.md for more information.

Python 3.7.2 (default, Jan 29 2022, 00:14:36) [GCC 4.8.5 20150623 (Red Hat 4.8.5-39)]

PLATFORM: Linux dongfei 3.10.0-1062.el7.x86_64 #1 SMP Wed Aug 7 18:08:02 UTC 2019 x86_64 x86_64

PYTHON LIBS: GetOrganelleLib 1.7.5.3; numpy 1.21.5; sympy 1.9; scipy 1.7.3

DEPENDENCIES: Bowtie2 2.4.5; SPAdes 3.15.3; Blast 2.12.0

GETORG_PATH=/software/GetOrganelle/GetOrganelleLib

SEED DB: embplant_pt customized; embplant_mt customized

LABEL DB: embplant_pt customized; embplant_mt customized

WORKING DIR: /home/pengs/20220214_nuohe6DNA/X101SC21093499-Z02-J023/2.cleandata/PS-00372_FDSW220030817-1r

/software/GetOrganelle/get_organelle_from_reads.py -1 PS-00372_FDSW220030817-1r_1.clean.fq.gz -2 PS-00372_FDSW220030817-1r_2.clean.fq.gz -o cp_out -t 5 -R 15 -k 21,45,65,85,105 -F embplant_pt

2022-02-14 16:43:05,860 - INFO: Pre-reading fastq ...

2022-02-14 16:43:05,861 - INFO: Estimating reads to use ... (to use all reads, set '--reduce-reads-for-coverage inf --max-reads inf')

2022-02-14 16:43:05,936 - INFO: Tasting 100000+100000 reads ...

2022-02-14 16:43:29,437 - INFO: Tasting 500000+500000 reads ...

2022-02-14 16:44:00,730 - INFO: Estimating reads to use finished.

2022-02-14 16:44:00,732 - INFO: Unzipping reads file: PS-00372_FDSW220030817-1r_1.clean.fq.gz (1029289733 bytes)

2022-02-14 16:44:28,643 - INFO: Unzipping reads file: PS-00372_FDSW220030817-1r_2.clean.fq.gz (1110455311 bytes)

2022-02-14 16:44:54,929 - INFO: Counting read qualities ...

2022-02-14 16:44:55,061 - INFO: Identified quality encoding format = Sanger

2022-02-14 16:44:55,061 - INFO: Phred offset = 33

2022-02-14 16:44:55,062 - INFO: Trimming bases with qualities (0.00%): 33..33 !

2022-02-14 16:44:55,091 - INFO: Mean error rate = 0.003

2022-02-14 16:44:55,091 - INFO: Counting read lengths ...

2022-02-14 16:45:22,950 - INFO: Mean = 150.0 bp, maximum = 150 bp.

2022-02-14 16:45:22,950 - INFO: Reads used = 14807394+14807394

2022-02-14 16:45:22,950 - INFO: Pre-reading fastq finished.

2022-02-14 16:45:22,951 - INFO: Making seed reads ...

2022-02-14 16:45:22,997 - INFO: Making seed - bowtie2 index ...

2022-02-14 16:45:36,933 - INFO: Making seed - bowtie2 index finished.

2022-02-14 16:45:36,933 - INFO: Mapping reads to seed bowtie2 index ...

2022-02-14 16:47:53,648 - INFO: Mapping finished.

2022-02-14 16:47:53,648 - INFO: Seed reads made: cp_out/seed/embplant_pt.initial.fq (178612947 bytes)

2022-02-14 16:47:53,648 - INFO: Making seed reads finished.

2022-02-14 16:47:53,648 - INFO: Checking seed reads and parameters ...

2022-02-14 16:47:53,648 - INFO: The automatically-estimated parameter(s) do not ensure the best choice(s).

2022-02-14 16:47:53,648 - INFO: If the result graph is not a circular organelle genome,

2022-02-14 16:47:53,648 - INFO: you could adjust the value(s) of '-w'/'-R' for another new run.

2022-02-14 16:48:11,024 - INFO: Pre-assembling mapped reads ...

2022-02-14 16:48:31,878 - INFO: Pre-assembling mapped reads finished.

2022-02-14 16:48:31,878 - INFO: Estimated embplant_pt-hitting base-coverage = 663.20

2022-02-14 16:48:32,130 - INFO: Reads reduced to = 11163605+11163605

2022-02-14 16:48:32,130 - INFO: Adjusting expected embplant_pt base coverage to 500.00

2022-02-14 16:48:32,131 - INFO: Estimated word size(s): 112

2022-02-14 16:48:32,131 - INFO: Setting '-w 112'

2022-02-14 16:48:32,131 - INFO: Setting '--max-extending-len inf'

2022-02-14 16:48:32,859 - INFO: Checking seed reads and parameters finished.

2022-02-14 16:48:32,859 - INFO: Making read index ...

2022-02-14 16:49:30,014 - INFO: For cp_out/1-PS-00372_FDSW220030817-1r_1.clean.fq.gz.fastq, only top 11163605 reads are used in downstream analysis.

2022-02-14 16:50:32,543 - INFO: For cp_out/2-PS-00372_FDSW220030817-1r_2.clean.fq.gz.fastq, only top 11163605 reads are used in downstream analysis.

2022-02-14 16:50:47,042 - INFO: 18284933 candidates in all 22327210 reads

2022-02-14 16:50:47,042 - INFO: Pre-grouping reads ...

2022-02-14 16:50:47,042 - INFO: Setting '--pre-w 112'

2022-02-14 16:50:48,521 - INFO: 200000/3216120 used/duplicated

2022-02-14 16:51:06,617 - INFO: 1404 groups made.

2022-02-14 16:51:11,606 - INFO: Making read index finished.

2022-02-14 16:51:11,606 - INFO: Extending ...

2022-02-14 16:51:11,606 - INFO: Adding initial words ...

2022-02-14 16:51:24,072 - INFO: AW 4799304

2022-02-14 16:52:57,994 - INFO: Round 1: 18284933/18284933 AI 240610 AW 5053566

2022-02-14 16:54:29,895 - INFO: Round 2: 18284933/18284933 AI 244108 AW 5074420

2022-02-14 16:55:57,407 - INFO: Round 3: 18284933/18284933 AI 244263 AW 5077648

2022-02-14 16:57:33,879 - INFO: Round 4: 18284933/18284933 AI 244388 AW 5080098

2022-02-14 16:59:03,965 - INFO: Round 5: 18284933/18284933 AI 244494 AW 5082178

2022-02-14 17:00:27,076 - INFO: Round 6: 18284933/18284933 AI 244559 AW 5083382

2022-02-14 17:01:50,159 - INFO: Round 7: 18284933/18284933 AI 244612 AW 5084446

2022-02-14 17:03:14,765 - INFO: Round 8: 18284933/18284933 AI 244653 AW 5085146

2022-02-14 17:04:38,475 - INFO: Round 9: 18284933/18284933 AI 244677 AW 5085728

2022-02-14 17:06:01,534 - INFO: Round 10: 18284933/18284933 AI 244706 AW 5086196

2022-02-14 17:07:24,759 - INFO: Round 11: 18284933/18284933 AI 244724 AW 5086534

2022-02-14 17:08:47,960 - INFO: Round 12: 18284933/18284933 AI 244747 AW 5086900

2022-02-14 17:10:11,344 - INFO: Round 13: 18284933/18284933 AI 244757 AW 5087004

2022-02-14 17:11:34,986 - INFO: Round 14: 18284933/18284933 AI 244781 AW 5087464

2022-02-14 17:12:56,421 - INFO: Round 15: 18284933/18284933 AI 244796 AW 5087696

2022-02-14 17:12:56,421 - INFO: Hit the round limit 15 and terminated ...

2022-02-14 17:13:22,228 - INFO: Extending finished.

2022-02-14 17:13:24,265 - INFO: Separating extended fastq file ...

2022-02-14 17:13:25,386 - INFO: Setting '-k 21,45,65,85,105'

2022-02-14 17:13:25,386 - INFO: Assembling using SPAdes ...

2022-02-14 17:13:25,447 - INFO: spades.py -t 5 --phred-offset 33 -1 cp_out/extended_1_paired.fq -2 cp_out/extended_2_paired.fq --s1 cp_out/extended_1_unpaired.fq --s2 cp_out/extended_2_unpaired.fq -k 21,45,65,85,105 -o cp_out/extended_spades

2022-02-14 17:14:24,258 - INFO: Insert size = 356.124, deviation = 74.5816, left quantile = 263, right quantile = 452

2022-02-14 17:14:24,259 - INFO: Assembling finished.

2022-02-14 17:14:37,186 - INFO: Slimming cp_out/extended_spades/K105/assembly_graph.fastg finished!

2022-02-14 17:14:37,186 - INFO: Slimming assembly graphs finished.

2022-02-14 17:14:37,187 - INFO: Extracting embplant_pt from the assemblies ...

2022-02-14 17:14:37,188 - INFO: Disentangling cp_out/extended_spades/K105/assembly_graph.fastg.extend-embplant_pt-embplant_mt.fastg as a circular genome ...

2022-02-14 17:14:37,274 - INFO: Vertex_307 #copy = 1

2022-02-14 17:14:37,274 - INFO: Vertex_609 #copy = 1

2022-02-14 17:14:37,274 - INFO: Vertex_629_549 #copy = 2

2022-02-14 17:14:37,274 - INFO: Average embplant_pt kmer-coverage = 128.8

2022-02-14 17:14:37,274 - INFO: Average embplant_pt base-coverage = 420.1

2022-02-14 17:14:37,274 - INFO: Writing output ...

2022-02-14 17:14:37,321 - WARNING: More than one circular genome structure produced ...

2022-02-14 17:14:37,321 - WARNING: Please check the final result to confirm whether they are simply different in SSC direction (two flip-flop configurations)!

2022-02-14 17:14:37,874 - INFO: Detecting large repeats (>1000 bp) in PATH1 with IRs detected, Total:LSC:SSC:Repeat(bp) = 158571:87289:18940:26171

2022-02-14 17:14:37,874 - INFO: Writing PATH1 of complete embplant_pt to cp_out/embplant_pt.K105.complete.graph1.1.path_sequence.fasta

2022-02-14 17:14:37,875 - INFO: Writing PATH2 of complete embplant_pt to cp_out/embplant_pt.K105.complete.graph1.2.path_sequence.fasta

2022-02-14 17:14:37,875 - INFO: Writing GRAPH to cp_out/embplant_pt.K105.complete.graph1.selected_graph.gfa

2022-02-14 17:14:37,933 - INFO: Result status of embplant_pt: circular genome

2022-02-14 17:14:38,096 - INFO: Please visualize cp_out/extended_K105.assembly_graph.fastg.extend-embplant_pt-embplant_mt.fastg using Bandage to confirm the final result.

2022-02-14 17:14:38,096 - INFO: Writing output finished.

2022-02-14 17:14:38,097 - INFO: Extracting embplant_pt from the assemblies finished.

Total cost 1897.80 s

Thank you!
