# Supplementary material for: Comparative chloroplast genome analysis of Sambucus L. (Viburnaceae): inference for phylogenetic relationships among the closely related Sambucus adnata Wall. ex DC Sambucus javanica Blume
Source: Front Plant Sci. 2023 Jun 16;14:1179510. doi: 10.3389/fpls.2023.1179510 (PMC10313135; doi:10.3389/fpls.2023.1179510)
Supplement: Supplementary file 4 [file Table_2.docx]

Supplementary Material

**Table S1** Taxonomic status of *Sambucus* species.

| **ID** | **Taxa** | **Taxonomic/Nomenclatural status** | **Confidence level** | **Source** | **Source ID** | **IPNI ID** |
| --- | --- | --- | --- | --- | --- | --- |
|  |  | **TPL** |  |  |  |  |
| kew-2486600 | *S. acuminata* Greene | Unresolved | L | WCSP (in review) | 623367 | 227131-2 |
| tro-6000746 | *S. adnata* Wall. ex DC. | Accepted | M | TRO | 6000746 |  |
| kew-2486602 | *S. africana* Standl. | Unresolved | L | WCSP (in review) | 623369 | 149285-1 |
| kew-2486580 | *S. alba* Raf. | Unresolved | L | WCSP (in review) | 623347 | 149286-1 |
| kew-2486597 | *S. angustifolia* Noronha | Unresolved | L | WCSP (in review) | 623364 | 149287-1 |
| kew-2486574 | *S. arborescens* Gilib. | Unresolved | L | WCSP (in review) | 623341 | 149289-1 |
| kew-2486595 | *arborescens* Nutt. | Unresolved | L | WCSP (in review) | 623362 |  |
| kew-2486576 | *S. arisanensis* Kaneh. & Sasaki | Unresolved | L | WCSP (in review) | 623343 |  |
| kew-2486577 | *S. aurea* Carrière | Unresolved | L | WCSP (in review) | 623344 | 149293-1 |
| tro-50044259 | *S. australasica* (Lindl.) Fritsch | Accepted | M | TRO | 50044259 | 149294-1 |
| tro-6000522 | *S. australis* Cham. & Schltdl. | Accepted | M | TRO | 6000522 | 149295-1 |
| kew-2486583 | *S. borealis* Greene | Unresolved | L | WCSP (in review) | 623350 | 227133-2 |
| kew-2486585 | *S. californica* K.Koch | Unresolved | L | WCSP (in review) | 623352 | 321981-2 |
| tro-6000146 | *S. callicarpa* Greene | Accepted | M | TRO | 6000146 | 289824-2 |
| kew-2486578 | *S. canadensis* L. | Accepted | M | WCSP (in review) | 623345 | 321978-2 |
| tro-6000148 | *S. cerulea* Raf. | Accepted | M | TRO | 6000148 |  |
| tro-6000290 | *S. Cerulea var. neomexicana* Rehder | Accepted | L | TRO | 6000290 |  |
| kew-2486624 | *S. cochinchinensis* Spreng | Unresolved | L | WCSP (in review) | 623391 | 149307-1 |
| kew-2486625 | *S. columnaris* Lebas | Unresolved | L | WCSP (in review) | 623392 | 149308-1 |
| tro-6000493 | *S. coreana* Kom. & Aliss. | Unresolved | L | TRO | 6000493 |  |
| kew-2486626 | *S. coriacea* Greene | Unresolved | L | WCSP (in review) | 623393 | 227157-2 |
| kew-2486627 | *S. decipiens* M.E.Jones | Unresolved | L | WCSP (in review) | 623394 | 227158-2 |
| kew-2486636 | *S. dimidiata* Raf. | Unresolved | L | WCSP (in review) | 623403 | 149311-1 |
| kew-2486629 | *S. dissecta* K.Koch | Unresolved | L | WCSP (in review) | 623396 | 149312-1 |
| kew-2486621 | *S. eberhardtii* Danguy | Unresolved | L | WCSP (in review) | 623388 | 149313-1 |
| kew-2486631 | *S. ebuloides* Desv. ex DC. | Unresolved | L | WCSP (in review) | 623398 | 149314-1 |
| kew-2486632 | *S. ebulus* L. | Accepted | M | WCSP (in review) | 623399 | 149315-1 |
| tro-50009470 | *Ebulus subsp. africana* (Engl.) Bolli | Accepted | L | TRO | 50009470 | 978601-1 |
| kew-2486634 | *S. elegans* K.Koch | Unresolved | L | WCSP (in review) | 623401 | 149316-1 |
| kew-2486635 | *S. ferax* A.Nelson | Unresolved | L | WCSP (in review) | 623402 | 227160-2 |
| kew-2486613 | *S. fimbriata* Greene | Unresolved | L | WCSP (in review) | 623380 | 227161-2 |
| kew-2486630 | *S. floribunda* K.Koch | Unresolved | L | WCSP (in review) | 623397 |  |
| kew-2486628 | *S. florida* Salisb. | Unresolved | L | WCSP (in review) | 623395 | 149320-1 |
| kew-2486607 | *S. fontenayi* K.Koch ex Dippel | Unresolved | L | WCSP (in review) | 623374 | 149322-1 |
| kew-2486608 | *S. fontenaysii* Carrière | Unresolved | L | WCSP (in review) | 623375 | 149323-1 |
| kew-2486610 | *S. gaudichaudiana* DC. | Unresolved | L | WCSP (in review) | 623377 | 149325-1 |
| kew-2486612 | *S. glabrescens* Nakai | Unresolved | L | WCSP (in review) | 623379 | 149327-1 |
| kew-2486605 | *S. glauca* Benth. | Unresolved | L | WCSP (in review) | 623372 | 149328-1 |
| kew-2486614 | *S. glauca* Nutt. | Unresolved | L | WCSP (in review) | 623381 | 227162-2 |
| kew-2486618 | *S. hirta* Tausch | Unresolved | L | WCSP (in review) | 623385 | 149332-1 |
| kew-2486586 | *S. integerrima* Stokes | Unresolved | L | WCSP (in review) | 623353 | 149336-1 |
| kew-2486525 | *S. intermedia* Carrière | Unresolved | L | WCSP (in review) | 623292 | 149337-1 |
| tro-50111461 | *S. javanica* Blume | Accepted | M | TRO | 50111461 |  |
| kew-2486539 | *S. kamtschatica* E.L.Wolf | Unresolved | L | WCSP (in review) | 623306 | 149341-1 |
| kew-2486531 | *S. laciniata* Mill. | Unresolved | L | WCSP (in review) | 623298 | 149342-1 |
| kew-2486523 | *S. lanceolata* R.Br. | Accepted | M | WCSP (in review) | 623290 |  |
| kew-2486533 | *S. latipinna* Nakai | Unresolved | L | WCSP (in review) | 623300 | 149344-1 |
| kew-2486535 | *S. leiosperma* Leiberg | Unresolved | L | WCSP (in review) | 623302 | 227168-2 |
| kew-2486536 | *S. leucocarpa* K.Koch | Unresolved | L | WCSP (in review) | 623303 | 149346-1 |
| kew-2486537 | *S. linearis* K.Koch | Unresolved | L | WCSP (in review) | 623304 |  |
| kew-2486515 | *S. longipes* Nakai | Unresolved | L | WCSP (in review) | 623282 |  |
| kew-2587856 | *S. loureiroana* DC. | Unresolved | L | WCSP (in review) | 721390 | 149349-1 |
| kew-2486530 | *S. lucida* Tausch | Unresolved | L | WCSP (in review) | 623297 | 149350-1 |
| kew-2486510 | *S. maritima* Greene | Unresolved | L | WCSP (in review) | 623277 | 227169-2 |
| kew-2486511 | *S. medullosa* Gilib. | Unresolved | L | WCSP (in review) | 623278 | 149354-1 |
| tro-6000149 | *S. melanocarpa* A. Gray | Accepted | M | TRO | 6000149 | 227170-2 |
| tro-6000294 | *S. microbotrys* Rydb. | Accepted | M | TRO | 6000294 | 227182-2 |
| kew-2486507 | *S. microsperma* Nakai | Unresolved | L | WCSP (in review) | 623274 |  |
| kew-2486516 | *S. miquelii* (Nakai) Kom. | Unresolved | L | WCSP (in review) | 623283 |  |
| kew-2486517 | *S. monstrosa* Loudon | Unresolved | L | WCSP (in review) | 623284 | 149360-1 |
| tro-50102530 | *Neomexicana var. vestita* (Wooton & Standl.) Kearney & Peebles | Unresolved | L | TRO | 50102530 | 227184-2 |
| kew-2486519 | *S. nigra* L. | Accepted | M | WCSP (in review) | 623286 | 30122169-2 |
| tro-6000151 | *S. orbiculata* Greene | Accepted | M | TRO | 6000151 | 227186-2 |
| kew-2486569 | *S. palmensis* Link | Accepted | M | WCSP (in review) | 623336 |  |
| kew-2486570 | *S. paucijuga* Steven | Unresolved | L | WCSP (in review) | 623337 | 149369-1 |
| kew-2486548 | *S. pendula* Nakai | Unresolved | L | WCSP (in review) | 623315 | 149370-1 |
| tro-6000196 | *S. peruviana* Kunth | Accepted | M | TRO | 6000196 | 321979-2 |
| kew-2486542 | *S. phyteumoides* DC. | Unresolved | L | WCSP (in review) | 623309 | 149374-1 |
| kew-2486543 | *S. planteriensis* Simon-Louis ex Dippel | Unresolved | L | WCSP (in review) | 623310 | 149375-1 |
| kew-2486544 | *S. plantierensis* Koehne | Unresolved | L | WCSP (in review) | 623311 | 149376-1 |
| kew-2486545 | *S. plumosa* Carrière | Unresolved | L | WCSP (in review) | 623312 | 149377-1 |
| kew-2486547 | *S. pubens* Michx. | Accepted | M | WCSP (in review) | 623314 | 227191-2 |
| kew-2486549 | *S. pubescens* Pers. | Unresolved | L | WCSP (in review) | 623316 | 149380-1 |
| kew-2486550 | *S. pulverulenta* K.Koch | Unresolved | L | WCSP (in review) | 623317 | 149381-1 |
| kew-2486551 | *S. pyramidata* Lebas | Unresolved | L | WCSP (in review) | 623318 | 149382-1 |
| kew-2486552 | *S. racemosa* L. | Accepted | M | WCSP (in review) | 623319 | 30056767-2 |
| tro-6000329 | *Racemosa f. dissecta*(Britton) Scoggan | Accepted | L | TRO | 6000329 | 227210-2 |
| tro-6000152 | *Racemosa var. leucocarpa* (Torr. & A. Gray) Cronquist | Accepted | L | TRO | 6000152 | 227215-2 |
| tro-6000327 | *Racemosa f. rosaeflora* (Carrière) Scoggan | Accepted | L | TRO | 6000327 | 227223-2 |
| tro-50111487 | *S. rehderana* Schwer. | Unresolved | L | TRO | 50111487 | 227228-2 |
| kew-2485711 | *S. repens* Raf. | Unresolved | L | WCSP (in review) | 622487 | 149385-1 |
| kew-2484934 | *S. rotundifolia* Lodd. ex Sweet | Unresolved | L | WCSP (in review) | 621713 | 149386-1 |
| kew-2485966 | *S. rubra* Buch. -Ham. ex Wall. | Unresolved | L | WCSP (in review) | 622738 | 149387-1 |
| kew-2485193 | *S. rupestris* Raf. | Unresolved | L | WCSP (in review) | 621972 | 149388-1 |
| kew-2485194 | *S. sachalinensis* Pojark. | Unresolved | L | WCSP (in review) | 621973 | 149389-1 |
| kew-2485196 | *S. seminata* Schwer. | Unresolved | L | WCSP (in review) | 621975 | 227231-2 |
| kew-2485197 | *S. sibirica* Nakai | Accepted | M | WCSP (in review) | 621976 | 149392-1 |
| tro-100374044 | *S. sieboldiana* (Miq.) Blume ex Schwer. | Accepted | M | TRO | 100374044 | 149394-1 |
| tro-6000500 | *S. Sieboldiana var. pinnatisecta* G.Y. Luo & P.H. Huang | Accepted | L | TRO | 6000500 |  |
| kew-2605419 | *S. strumpfii* Gutte | Unresolved | L | WCSP (in review) | 738551 |  |
| kew-2485203 | *S. suaveolens* DC. | Unresolved | L | WCSP (in review) | 621982 | 149396-1 |
| kew-2485204 | *S. sylvestris* Bubani | Unresolved | L | WCSP (in review) | 621983 | 149397-1 |
| kew-2485205 | *S. thunbergiana* Blume ex Miq. | Unresolved | L | WCSP (in review) | 621984 | 149398-1 |
| kew-2485183 | *S. thunbergii* G.Don | Unresolved | L | WCSP (in review) | 621962 | 149399-1 |
| kew-2605420 | *S. tigranii* Troitsky | Unresolved | L | WCSP (in review) | 738552 | 77085402-1 |
| kew-2485200 | *S. tiliifolia* Wall. ex DC. | Unresolved | L | WCSP (in review) | 621979 |  |
| kew-2490699 | *S. trifida* A.Heller | Unresolved | L | WCSP (in review) | 627434 | 227233-2 |
| tro-6000154 | *S. velutina* Durand & Hilg. | Accepted | M | TRO | 6000154 | 227234-2 |
| kew-2485178 | *S. verrucosa* Raf. | Unresolved | L | WCSP (in review) | 621957 | 149403-1 |
| kew-2485180 | *S. virescens* Desf. | Unresolved | L | WCSP (in review) | 621959 | 149405-1 |
| tro-50044266 | *S. viridis* Petrov | Unresolved | L | TRO | 50044266 |  |
| kew-2485190 | *S. vulgaris* Neck. | Unresolved | L | WCSP (in review) | 621969 |  |
| tro-50009472 | *S. wightiana* Wall. ex Wight & Arn. | Accepted | M | TRO | 50009472 |  |
| tro-6000520 | *S. williamsii* Hance | Accepted | M | TRO | 6000520 | 149409-1 |
| kew-2485186 | *S. xanthocarpa* F.Muell. | Unresolved | L | WCSP (in review) | 621965 | 149410-1 |
