# Supplementary material for: Comparative chloroplast genome analysis of Sambucus L. (Viburnaceae): inference for phylogenetic relationships among the closely related Sambucus adnata Wall. ex DC Sambucus javanica Blume
Source: Front Plant Sci. 2023 Jun 16;14:1179510. doi: 10.3389/fpls.2023.1179510 (PMC10313135; doi:10.3389/fpls.2023.1179510)
Supplement: Supplementary file 5 [file Table_3.docx]

Supplementary Material

**Table S2** Specimens used in the present study.

|  | **Category** | **Species** | **Voucher Number** | **Accession Number** | **Locality** |
| --- | --- | --- | --- | --- | --- |
| **1.** | **Newly sequenced plastomes** |  |  |  |  |
|  | *Sambucus* L. | *Sambucus javanica* | DBS0055 | OM868260 | Ta-pieh mountains, China |
|  |  | *Sambucus javanica* | CXZ2 | ON006397 | Xin County, Henan  Province, China. |
|  |  | *Sambucus javanica* | ZSY0003 | ON006398 | Qianshan City,  Anhui Province,  China. |
|  |  | *Sambucus javanica* (S. chinensis var. pinnatilobatus G. W. Hu) | HGW-M-261 | ON006402 | Hunan, China. |
|  |  | *Sambucus williamsii* | HJD1137 | OM937121 | Ta-pieh mountains, China |
|  |  | *Sambucus canadensis* | SAJIT004036 | OM937119 | Mt. Kenya, Irangi Forest. |
|  |  | *Sambucus canadensis* | SAJIT003951 | OM937120 | Meru, Kenya. |
|  |  | *Sambucus adnata* | PS-00372 | ON006399 | Zayu County, Tibet, China. |
|  |  | *Sambucus adnata* | Wbgd-140 | ON006400 | Yuanmou County,  Yunnan Province,  China. |
|  |  | *Sambucus adnata* | YJX-0234 | ON006401 | Kangding City,  Sichuan Province,  China. |
| **2.** | **Plastomes obtained from GenBank** |  |  |  |  |
|  | **a.** In-groups |  |  |  |  |
|  | *Adoxa* L. (*Tetradoxa/ Sinadoxa*) | *Adoxa moschatellina* | - | NC_034792 | - |
|  |  | *Adoxa omeiensis* (*Tetradoxa omeiensis*) | - | NC_034793 | - |
|  |  | *Adoxa corydalifolia* (*Sinadoxa corydalifolia*) | - | NC_032040 | - |
|  | *Sambucus* L. | *Sambucus nigra* | - | NC_045061 | - |
|  |  | *Sambucus nigra* | - | MN524612 | - |
|  |  | *Sambucus williamsii* | - | NC_033878 | - |
|  |  | *Sambucus chinensis* (*S. javanica*) | - | MW455170 | - |
|  |  | *Sambucus chinensis* (*S. javanica*) | - | OK065821 | - |
|  | *Viburnum* L. | *Viburnum awabuki* | - | MT507590 | - |
|  |  | *Viburnum betulifolium* | - | NC_037951 | - |
|  |  | *Viburnum brachybotryum* | - | NC_045062 | - |
|  |  | *Viburnum burejaeticum* | - | MT507599 | - |
|  |  | *Viburnum cinnamomifolium* | - | MT507596 | - |
|  |  | *Viburnum cylindricum* | - | MK397932 | - |
|  |  | *Viburnum erosum* | - | MN641480 | - |
|  |  | *Viburnum farreri* | - | MT507601 | - |
|  |  | *Viburnum hanceanum* | - | MT507603 | - |
|  |  | *Viburnum henryi* | - | MT507592 | - |
|  |  | *Viburnum luzonicum* | - | MT507589 | - |
|  |  | *Viburnum melanocarpum* | - | MT507587 | - |
|  |  | *Viburnum nervosum* | - | MT507598 | - |
|  |  | *Viburnum setigerum* | - | MT507585 | - |
|  |  | *Viburnum oliganthum* | - | MT507602 | - |
|  |  | *Viburnum opulus* | - | MT507605 | - |
|  |  | *Viburnum propinquum* | - | MT507593 | - |
|  |  | *Viburnum rhytidophyllum* | - | MT507594 | - |
|  |  | *Viburnum schensianum* | - | MT507600 | - |
|  |  | *Viburnum ternatum* | - | MT507595 | - |
|  |  | *Viburnum utile* | - | NC_032296 | - |
|  |  | *Viburnum fordiae* | - | MN524625 | - |
|  | a. Outgroups |  | - |  | - |
|  | Araliaceae | *Eleutherococcus gracilistylus* | - | KT153020 | - |
|  |  | *Panax ginseng* | - | MH049735 | - |
