# Supplementary material for: Comparative chloroplast genome analysis of Sambucus L. (Viburnaceae): inference for phylogenetic relationships among the closely related Sambucus adnata Wall. ex DC Sambucus javanica Blume
Source: Front Plant Sci. 2023 Jun 16;14:1179510. doi: 10.3389/fpls.2023.1179510 (PMC10313135; doi:10.3389/fpls.2023.1179510)
Supplement: Supplementary file 6 [file Table_4.docx]

Supplementary Material

**Table S3** Repeat element characterization

1. Total number of identified SSRs.

|  | **Simple Sequence Repeats (SSRs)** | | | | | |  |
| --- | --- | --- | --- | --- | --- | --- | --- |
| **Taxon** | Mono | Di | Tri | Tetra | Penta | Hexa | Total |
| OM937121_*S. williamsii* | 48 | 7 | 3 | 8 | 0 | 2 | 68 |
| NC_033878_*S. williamsii* | 43 | 6 | 3 | 8 | 2 | 0 | 62 |
| OM937119_*S. canadensis* | 46 | 8 | 2 | 7 | 1 | 0 | 64 |
| OM937120_*S. canadensis* | 46 | 8 | 2 | 7 | 1 | 0 | 64 |
| ON006401_*S. adnata* | 34 | 9 | 2 | 6 | 1 | 0 | 52 |
| ON006400_*S. adnata* | 33 | 9 | 2 | 6 | 2 |  | 52 |
| ON006399_*S. adnata* | 33 | 9 | 3 | 6 | 2 | 0 | 53 |
| MW007718_*S. adnata* | 31 | 8 | 2 | 6 | 2 | 0 | 49 |
| ON006397_*S. javanica* | 32 | 10 | 2 | 6 | 1 | 0 | 51 |
| OM868260_*S. javanica* | 32 | 9 | 2 | 6 | 1 | 0 | 50 |
| ON006398_*S. javanica* | 32 | 9 | 2 | 5 | 1 | 0 | 49 |
| ON006402_*S. chinensis* var. *pinnatilobatus* | 32 | 9 | 2 | 6 | 1 | 0 | 50 |

1. Positions of repeat elements.

| **Repeat size (bp)** | **1st start** | **Repeat type** | **2nd start** | **Location 1** | **Location 2** | **Region** |
| --- | --- | --- | --- | --- | --- | --- |
| 44 | 77417 | P | 77417 | psaB | *PsaA* | LSC |
| 40 | 117041 | F | 117080 | psaB | *PsaA* | LSC |
| 46 | 94632 | F | 94650 | *IGS (petN-psbM)* | IGS (*petN-psbM*) | LSC |
| 46 | 94632 | P | 151396 | psbB | *psbB* | LSC |
| 46 | 94650 | P | 151414 | *IGS (psbE-petL)* | *rpl2*0 | LSC |
| 46 | 151396 | F | 151414 | *IGS (rrn5-trnR-ACG)* | *IGS (rrn5-trnR-CG)* | IRb |
| 39 | 45607 | F | 101732 | Ycf2 | *Ycf2* | IRa / IRb |
| 39 | 45607 | F | 124437 | *Ycf*2 | Ycf2 | IRa/ IRb |
| 39 | 45607 | P | 144321 | *Ycf*2 | *Ycf2* | IRb/IRa |
| 42 | 101730 | F | 79141 | *petD* | *petD* | LSC |
| 42 | 124435 | P | 144320 | *rrn23* | *rrn23* | IRb |
| 41 | 40578 | F | 42802 | *rrn23* | *Ycf*2 | IRb/IRa |
| 30 | 9015 | P | 47336 | *rrn23* | *Ycf*2 | IRb/IRa |
| 30 | 94644 | F | 94662 | *IGS (rrn5-trnR-ACG)* | IGS (*rrn5-trnR-ACG)* | IRb |
| 30 | 94644 | P | 151400 | *IGS (psbk-psbI)* | *Ycf3* | LSC |
| 30 | 94662 | P | 151418 | *IGS (trnS-GCU-* *trnG-UCC)* | *trnG-UCC* | LSC |
| 35 | 45610 | F | 98643 | IGS (trnC-GCU- PetN) | IGS (*trnC-GCU- PetN*) | LSC |
| 35 | 45610 | F | 147414 | IGS (psbk-psbI) | p*sbC* | LSC |
| 31 | 48730 | F | 48744 | *rps*12 | *IGS* (*trnV-GAC- rps12*) | LSC/IRA |
| 32 | 9013 | F | 37381 | psaB | *psaA* | LSC |
| 31 | 62147 | P | 62147 | psaB | *psaA* | LSC |
| 30 | 37383 | P | 47336 | *IGS (petN-psbM)* | *IGS (petN-psbM)* | LSC |
| 30 | 45608 | P | 78705 | *psaB* | *psaB* | LSC |
| 30 | 92199 | F | 92241 | *IGS (psbE-petL)* | *rpl20* | LSC |
| 30 | 92199 | P | 153821 | *IGS (rrn5-trnR-ACG)* | *IGS (rrn5-trnR-CG)* | IRb |
| 30 | 92241 | P | 153863 | *IGS (rrn5-trnR-ACG)* | *IGS (trnN-GUU- trnR-ACG)* | IRb/IRa |
| 30 | 102754 | C | 143309 | *IGS (trnN-GUU- trnR-ACG)* | *IGS (trnN-GUU- trnR-ACG)* | IRb |
| 30 | 153821 | F | 153863 | *rps12* | *ndhA* | IRa |
