# Supplementary material for: Comparative chloroplast genome analysis of Sambucus L. (Viburnaceae): inference for phylogenetic relationships among the closely related Sambucus adnata Wall. ex DC Sambucus javanica Blume
Source: Front Plant Sci. 2023 Jun 16;14:1179510. doi: 10.3389/fpls.2023.1179510 (PMC10313135; doi:10.3389/fpls.2023.1179510)
Supplement: Supplementary file 7 [file Table_5.docx]

Supplementary Material

**Table S4** Nucleotide diversity of the *Sambucus* protein-coding genes

| **Gene** | ***S. williamsii* (OM937121)** | ***S. adnata* (ON006400)** | ***S. canadensis* (OM937119)** | ***S. nigra* (NC-045061)** | ***S. javanica* (OM868260)** |
| --- | --- | --- | --- | --- | --- |
| *accD* | 0.0013 | 0.00065 | 0.0013 | 0.0013 | 0 |
| *atpA* | 0.00328 | 0.00066 | 0.00394 | 0.00394 | 0 |
| *atpB* | 0.00134 | 0.00067 | 0.00067 | 0.00067 | 0 |
| *atpE* | 0.00498 | 0 | 0.00498 | 0.00498 | 0 |
| *atpF* | 0.00541 | 0.00721 | 0.00541 | 0 | 0 |
| *atpH* | 0 | 0 | 0 | 0 | 0 |
| *atpI* | 0.00134 | 0.00269 | 0.00134 | 0.00134 | 0 |
| *ccsA* | 0.00309 | 0.00206 | 0.00412 | 0.00412 | 0 |
| *cemA* | 0.0029 | 0 | 0.0029 | 0.0029 | 0 |
| *clpP* | 0 | 0 | 0 | 0.0017 | 0 |
| *infA* | 0 | 0 | 0 | 0 | 0 |
| *matK* | 0.0033 | 0.00132 | 0.00264 | 0.00264 | 0 |
| *ndhA* | 0.00183 | 0.00092 | 0.00275 | 0.00275 | 0 |
| *ndhB* | 0 | 0 | 0 | 0.0013 | 0 |
| *ndhC* | 0.00275 | 0.00275 | 0.00275 | 0.00275 | 0 |
| *ndhD* | 0.004 | 0.00067 | 0.00467 | 0.00466 | 0 |
| *ndhE* | 0.00327 | 0.00654 | 0.00327 | 0.00327 | 0 |
| *ndhF* | 0.00447 | 0.00178 | 0.00537 | 0.00537 | 0 |
| *ndhG* | 0 | 0.00188 | 0.00188 | 0.00188 | 0 |
| *ndhH* | 0.00169 | 0 | 0.00254 | 0.00254 | 0 |
| *ndhI* | 0.00198 | 0.00198 | 0.00198 | 0.00198 | 0 |
| *ndhJ* | 0 | 0 | 0 | 0 | 0 |
| *ndhK* | 0.00295 | 0 | 0.00295 | 0.00295 | 0 |
| *petA* | 0.00104 | 0 | 0 | 0 | 0 |
| *petB* | 0 | 0 | 0.00154 | 0.00154 | 0 |
| *petD* | 0.00417 | 0.00417 | 0.00833 | 0.01042 | 0 |
| *petG* | 0 | 0 | 0 | 0 | 0 |
| *petL* | 0.01042 | 0 | 0 | 0 | 0 |
| *petN* | 0 | 0 | 0 | 0 | 0 |
| *psaA* | 0.00133 | 0.00044 | 0.00089 | 0 | 0 |
| *psaB* | 0.00272 | 0.00091 | 0.00136 | 0.00136 | 0 |
| *psaC* | 0 | 0 | 0 | 0 | 0 |
| *psaI* | 0 | 0 | 0 | 0 | 0 |
| *psaJ* | 0.00775 | 0 | 0.00775 | 0.00775 | 0 |
| *psbA* | 0.00188 | 0.00094 | 0 | 0 | 0 |
| *psbB* | 0.00065 | 0 | 0.00065 | 0.00065 | 0 |
| *psbC* | 0.00281 | 0.00211 | 0.00281 | 0.00281 | 0 |
| *psbD* | 0.00188 | 0 | 0.00094 | 0.00094 | 0 |
| *psbE* | 0 | 0 | 0 | 0 | 0 |
| *psbF* | 0 | 0 | 0 | 0 | 0 |
| *psbH* | 0.0045 | 0 | 0 | 0 | 0 |
| *psbI* | 0 | 0 | 0 | 0 | 0 |
| *psbJ* | 0.00813 | 0 | 0.00813 | 0.00813 | 0 |
| *psbK* | 0 | 0 | 0 | 0 | 0 |
| *psbL* | 0 | 0 | 0 | 0 | 0 |
| *psbM* | 0.00952 | 0 | 0 | 0 | 0 |
| *psbN* | 0 | 0 | 0 | 0 | 0 |
| *psbT* | 0 | 0 | 0 | 0 | 0 |
| *psbZ* | 0 | 0 | 0 | 0 | 0 |
| *rbcL* | 0.0028 | 0.0028 | 0.0021 | 0.0021 | 0 |
| *rpl2* | 0 | 0 | 0 | 0 | 0 |
| *rpl14* | 0.00271 | 0 | 0.00271 | 0.00271 | 0 |
| *rpl16* | 0 | 0 | 0 | 0 | 0 |
| *rpl20* | 0 | 0 | 0.00258 | 0.00258 | 0 |
| *rpl22* | 0.00414 | 0 | 0.00414 | 0.00409 | 0 |
| *rpl23* | 0 | 0 | 0 | 0 | 0 |
| *rpl32* | 0 | 0 | 0 | 0 | 0 |
| *rpl33* | 0.01493 | 0 | 0.00995 | 0.00995 | 0 |
| *rpl36* | 0 | 0.00888 | 0 | 0 | 0 |
| *rpoA* | 0.00296 | 0.00197 | 0.00296 | 0.00296 | 0 |
| *rpoB* | 0.00187 | 0.00124 | 0.00249 | 0.00249 | 0 |
| *rpoC1* | 0.00097 | 0.00097 | 0.00193 | 0.00195 | 0 |
| *rpoC2* | 0.00336 | 0.0024 | 0.00312 | 0.00313 | 0 |
| *rps2* | 0 | 0 | 0.00141 | 0.00141 | 0 |
| *rps3* | 0.00304 | 0 | 0.00152 | 0.00152 | 0 |
| *rps4* | 0 | 0 | 0.00165 | 0.00165 | 0 |
| *rps7* | 0 | 0 | 0 | 0 | 0 |
| *rps8* | 0.00247 | 0.00494 | 0.00247 | 0.00247 | 0 |
| *rps11* | 0 | 0 | 0.00238 | 0.00238 | 0 |
| *rps12* | 0 | 0 | 0 | 0 | 0 |
| *rps14* | 0.00271 | 0 | 0 | 0.0033 | 0 |
| *rps15* | 0 | 0 | 0 | 0 | 0 |
| *rps16* | 0 | 0 | 0 | 0 | 0 |
| *rps18* | 0 | 0 | 0 | 0 | 0 |
| *rps19* | 0.00717 | 0.00358 | 0.00717 | 0.00717 | 0 |
| *ycf1* | 0.00665 | 0.00285 | 0.00475 | 0 | 0 |
| *ycf2* | 0.00058 | 0.00029 | 0.00058 | 0.00043 | 0 |
| *ycf3* | 0.00198 | 0.00198 | 0.00198 | 0.00198 | 0 |
| *ycf4* | 0.0018 | 0 | 0.00541 | 0.00541 | 0 |
| *ycf15* | 0 | 0 | 0 | 0 | 0 |
