# Supplementary material for: Comparative chloroplast genome analysis of Sambucus L. (Viburnaceae): inference for phylogenetic relationships among the closely related Sambucus adnata Wall. ex DC Sambucus javanica Blume
Source: Front Plant Sci. 2023 Jun 16;14:1179510. doi: 10.3389/fpls.2023.1179510 (PMC10313135; doi:10.3389/fpls.2023.1179510)
Supplement: Supplementary file 8 [file Table_6.docx]

Supplementary Material

**Table S5** Relative Synonymous Codon Usage (RSCU) and average percentage (%) RSCU analysis of *Sambucus* species.

1. *Sambucus canadensis*

| **Amino acid** | **Codons** | **Count *S. canadensis* (OM937120)** | **RSCU** | **% RSCU** | **Count *S. canadensis* (OM937119)** | **RSCU** | **% RSCU** | **Average % RSCU** |
| --- | --- | --- | --- | --- | --- | --- | --- | --- |
| Phenylalanine(F) | UUU | 2131 | 1.16 | 7.01 | 2131 | 1.16 | 6.96 | 6.99 |
|  | UUC | 1559 | 0.84 |  | 1536 | 0.84 |  |  |
| Leucine(L) | UUA | 1127 | 1.26 | 10.16 | 1140 | 1.29 | 10.04 | 10.10 |
|  | UUG | 1114 | 1.25 |  | 1118 | 1.27 |  |  |
|  | CUU | 1125 | 1.26 |  | 1118 | 1.27 |  |  |
|  | CUC | 676 | 0.76 |  | 635 | 0.72 |  |  |
|  | CUA | 794 | 0.89 |  | 778 | 0.88 |  |  |
|  | CUG | 514 | 0.58 |  | 501 | 0.57 |  |  |
| Isoleucine(I) | AUU | 1769 | 1.21 | 8.30 | 1815 | 1.23 | 8.40 | 8.35 |
|  | AUC | 1124 | 0.77 |  | 1134 | 0.77 |  |  |
|  | AUA | 1481 | 1.02 |  | 1476 | 693 |  |  |
| Methionine(M) | AUG | 916 | 1 | 1.73 | 947 | 1.00 | 1.80 | 1.77 |
| Valine(V) | GUU | 775 | 1.29 | 4.55 | 777 | 1.30 | 4.55 | 4.55 |
|  | GUC | 446 | 0.74 |  | 448 | 0.75 |  |  |
|  | GUA | 705 | 1.18 |  | 713 | 1.19 |  |  |
|  | GUG | 473 | 0.79 |  | 461 | 0.77 |  |  |
| Serine(S) | UCU | 1155 | 1.41 | 9.36 | 1169 | 1.42 | 9.40 | 9.38 |
|  | UCC | 924 | 1.13 |  | 897 | 1.09 |  |  |
|  | UCA | 987 | 1.2 |  | 1001 | 1.21 |  |  |
|  | UCG | 618 | 0.75 |  | 622 | 0.75 |  |  |
|  | AGU | 726 | 0.88 |  | 737 | 0.89 |  |  |
|  | AGC | 518 | 0.63 |  | 522 | 0.63 |  |  |
| Proline(P) | CCU | 634 | 1.02 | 4.71 | 661 | 1.16 | 4.68 | 4.70 |
|  | CCC | 621 | 1 |  | 600 | 0.97 |  |  |
|  | CCA | 790 | 1.27 |  | 773 | 1.26 |  |  |
|  | CCG | 438 | 0.71 |  | 429 | 0.70 |  |  |
| Threonine(T) | ACU | 636 | 1.1 | 5.25 | 671 | 1.16 | 4.40 | 4.83 |
|  | ACC | 615 | 1.07 |  | 591 | 1.02 |  |  |
|  | ACA | 693 | 1.2 |  | 693 | 1.20 |  |  |
|  | ACG | 360 | 0.63 |  | 361 | 0.62 |  |  |
| Alanine(A) | GCU | 460 | 1.21 | 2.88 | 483 | 1.25 | 2.94 | 2.91 |
|  | GCC | 389 | 1.03 |  | 403 | 1.04 |  |  |
|  | GCA | 437 | 1.15 |  | 430 | 1.11 |  |  |
|  | GCG | 230 | 0.61 |  | 234 | 0.60 |  |  |
| Tyrosine(Y) | UAU | 1437 | 1.35 | 4.05 | 1432 | 1.35 | 4.03 | 4.04 |
|  | UAC | 696 | 0.65 |  | 690 | 0.65 |  |  |
| Histidine(H) | CAU | 964 | 1.38 | 2.64 | 891 | 1.35 | 2.50 | 2.57 |
|  | CAC | 429 | 0.62 |  | 426 | 0.65 |  |  |
| Glutamine(Q) | CAA | 1100 | 1.4 | 2.98 | 1083 | 1.40 | 2.93 | 2.96 |
|  | CAG | 471 | 0.6 |  | 460 | 0.60 |  |  |
| Asparagine(N) | AAU | 1703 | 1.36 | 4.75 | 1742 | 0.39 | 4.77 | 4.76 |
|  | AAC | 800 | 0.64 |  | 769 | 0.61 |  |  |
| Lysine(K) | AAA | 2072 | 1.34 | 5.88 | 2111 | 1.36 | 5.90 | 5.89 |
|  | AAG | 1025 | 0.66 |  | 996 | 0.64 |  |  |
| Aspartic acid(D) | GAU | 1072 | 1.44 | 2.84 | 1111 | 1.45 | 2.90 | 2.87 |
|  | GAC | 422 | 0.56 |  | 417 | 0.55 |  |  |
| Glutamic acid (E) | GAA | 1381 | 1.38 | 3.80 | 1405 | 1.39 | 3.83 | 3.82 |
|  | GAG | 618 | 0.62 |  | 613 | 0.61 |  |  |
| Cysteine (C) | UGU | 691 | 1.23 | 2.13 | 683 | 1.23 | 2.11 | 2.12 |
|  | UGC | 429 | 0.77 |  | 426 | 0.77 |  |  |
| Tryptophan (W) | UGG | 734 | 1 | 1.39 | 784 | 1.00 | 1.49 | 1.44 |
| Arginine (R) | CGU | 370 | 0.64 | 6.55 | 387 | 0.66 | 6.64 | 6.60 |
|  | CGC | 271 | 0.47 |  | 265 | 0.45 |  |  |
|  | CGA | 628 | 1.09 |  | 641 | 1.10 |  |  |
|  | CGG | 444 | 0.77 |  | 435 | 0.75 |  |  |
|  | AGA | 1096 | 1.91 |  | 1127 | 1.93 |  |  |
|  | AGG | 641 | 1.11 |  | 641 | 1.10 |  |  |
| Glycine(G) | GGU | 570 | 1.02 | 4.26 | 595 | 1.02 | 4.45 | 4.36 |
|  | GGC | 364 | 0.65 |  | 369 | 0.63 |  |  |
|  | GGA | 749 | 1.33 |  | 801 | 1.37 |  |  |
|  | GGG | 562 | 1 |  | 579 | 0.99 |  |  |
| Stop codon (*) | UAA | 1121 | 1.13 | 5.64 | 1034 | 1.11 | 5.30 | 5.47 |
|  | UAG | 795 | 0.8 |  | 741 | 0.80 |  |  |
|  | UGA | 1055 | 1.07 |  | 1011 | 1.09 |  |  |
|  |  |  |  |  |  |  |  |  |
| Average codons |  | 52, 670 |  |  | 52, 670 |  |  |  |

b). *Sambucus williamsii*

| **Amino acid** | **Codon** | **Count *S. williamsii* (OM937121)** | **RSCU** | **%RSCU** | **Count *S. williamsii* (NC_033878)** | **RSCU** | **%RSCU** | **Average % RSCU** |
| --- | --- | --- | --- | --- | --- | --- | --- | --- |
| Phenylalanine(F) | UUU | 2131 | 1.16 | 6.96 | 2170 | 1.18 | 6.97 | 6.97 |
|  | UUC | 1541 | 0.84 |  | 1506 | 0.82 |  |  |
| Leucine(L) | UUA | 1139 | 1.26 | 10.30 | 1155 | 1.30 | 10.08 | 10.19 |
|  | UUG | 1105 | 1.22 |  | 1042 | 1.18 |  |  |
|  | CUU | 1076 | 1.19 |  | 1095 | 1.23 |  |  |
|  | CUC | 732 | 0.81 |  | 695 | 0.78 |  |  |
|  | CUA | 825 | 0,91 |  | 804 | 0.91 |  |  |
|  | CUG | 556 | 0.61 |  | 529 | 0.60 |  |  |
| Isoleucine(I) | AUU | 1757 | 1.22 | 8.20 | 1667 | 1.23 | 8.09 | 8.06 |
|  | AUC | 1156 | 0.8 |  | 1147 | 0.81 |  |  |
|  | AUA | 1413 | 0.98 |  | 1453 | 1.02 |  |  |
| Methionine(M) | AUG | 879 | 1 | 1.67 | 811 | 1.00 | 1.54 | 1.61 |
| Valine(V) | GUU | 814 | 133 | 4.64 | 794 | 1.28 | 4.71 | 4.68 |
|  | GUC | 453 | 0.74 |  | 448 | 0.72 |  |  |
|  | GUA | 729 | 1.19 |  | 782 | 1.26 |  |  |
|  | GUG | 452 | 0.74 |  | 459 | 0.74 |  |  |
| Serine(S) | UCU | 1172 | 1.43 | 9.31 | 1245 | 1.52 | 9.30 | 9.31 |
|  | UCC | 895 | 1.09 |  | 861 | 1.05 |  |  |
|  | UCA | 1044 | 1.27 |  | 1088 | 1.33 |  |  |
|  | UCG | 635 | 0.78 |  | 623 | 0.76 |  |  |
|  | AGU | 665 | 0.81 |  | 664 | 0.81 |  |  |
|  | AGC | 504 | 0,62 |  | 425 | 0.52 |  |  |
| Proline(P) | CCU | 671 | 1.06 | 4.82 | 663 | 1.03 | 4.87 | 4.85 |
|  | CCC | 596 | 0.94 |  | 627 | 0.97 |  |  |
|  | CCA | 820 | 1.29 |  | 845 | 1.32 |  |  |
|  | CCG | 456 | 0.72 |  | 437 | 0.68 |  |  |
| Threonine(T) | ACU | 634 | 1.13 | 4.25 | 606 | 1.08 | 4.27 | 4.26 |
|  | ACC | 564 | 1.01 |  | 598 | 1.06 |  |  |
|  | ACA | 645 | 1.15 |  | 700 | 1.24 |  |  |
|  | ACG | 401 | 0.71 |  | 350 | 0.62 |  |  |
| Alanine(A) | GCU | 429 | 1.16 | 2.80 | 495 | 1.25 | 3.00 | 2.90 |
|  | GCC | 352 | 0.96 |  | 365 | 0.92 |  |  |
|  | GCA | 458 | 1.24 |  | 488 | 1.23 |  |  |
|  | GCG | 235 | 0.64 |  | 233 | 0.59 |  |  |
| Tyrosine(Y) | UAU | 1427 | 1.31 | 4.13 | 1439 | 1.33 | 4.09 | 4.11 |
|  | UAC | 754 | 0.69 |  | 718 | 0.67 |  |  |
| Histidine(H) | CAU | 962 | 1.42 | 2.57 | 934 | 1.41 | 2.52 | 2.55 |
|  | CAC | 393 | 0.58 |  | 394 | 0.59 |  |  |
| Glutamine(Q) | CAA | 1119 | 1.4 | 3.02 | 1059 | 1.37 | 2.93 | 2.98 |
|  | CAG | 474 | 0.6 |  | 489 | 0.63 |  |  |
| Asparagine(N) | AAU | 1690 | 1.38 | 4.65 | 1684 | 1.38 | 4.64 | 4.65 |
|  | AAC | 766 | 0.62 |  | 765 | 0.62 |  |  |
| Lysine(K) | AAA | 2069 | 1.31 | 5.98 | 2117 | 1.34 | 6.07 | 6.03 |
|  | AAG | 1086 | 0.69 |  | 1087 | 0.66 |  |  |
| Aspartic acid(D) | GAU | 1058 | 1.41 | 2.83 | 1087 | 1.44 | 2.86 | 2.85 |
|  | GAC | 438 | 0.59 |  | 421 | 0.56 |  |  |
| Glutamic acid (E) | GAA | 1379 | 1.37 | 3.80 | 1416 | 1.41 | 3.82 | 3.81 |
|  | GAG | 629 | 0.63 |  | 599 | 0.59 |  |  |
| Cysteine (C) | UGU | 674 | 1.19 | 2.14 | 711 | 1.21 | 2.22 | 2.18 |
|  | UGC | 455 | 0.81 |  | 461 | 0.79 |  |  |
| Tryptophan (W) | UGG | 700 | 1 | 1.33 | 731 | 1.00 | 1.39 | 1.36 |
| Arginine (R) | CGU | 376 | 0.66 | 6.51 | 387 | 0.67 | 6.55 | 6.53 |
|  | CGC | 262 | 0.46 |  | 254 | 0.44 |  |  |
|  | CGA | 564 | 0.98 |  | 610 | 1.06 |  |  |
|  | CGG | 433 | 0.76 |  | 430 | 0.75 |  |  |
|  | AGA | 1178 | 2.06 |  | 1188 | 2.06 |  |  |
|  | AGG | 625 | 1.09 |  | 588 | 1.02 |  |  |
| Glycine(G) | GGU | 523 | 0.93 | 4.24 | 559 | 0.97 | 4.43 | 4.34 |
|  | GGC | 356 | 0.64 |  | 353 | 0.61 |  |  |
|  | GGA | 784 | 1.4 |  | 845 | 1.47 |  |  |
|  | GGG | 576 | 1.03 |  | 539 | 0.94 |  |  |
| Stop codon (*) | UAA | 1168 | 1.13 | 5.87 | 1092 | 1.07 | 5.83 | 5.85 |
|  | UAG | 837 | 0.81 |  | 811 | 0.79 |  |  |
|  | UGA | 1092 | 1.06 |  | 1172 | 1.14 |  |  |
|  |  |  |  |  |  |  |  |  |
| Average codons |  | 52, 778 |  |  | 52, 768 |  |  |  |

c). *Sambucus javanica*

| **Amino acid** | **Codon** | **Count**  ***S. javanica***  **(OM868260)** | **RSCU** | **%RSCU** | **Count**  ***S. javanica***  **(ON006397)** | **RSCU** | **%RSCU** | **Average % RSCU** |
| --- | --- | --- | --- | --- | --- | --- | --- | --- |
| Phenylalanine(F) | UUU | 2199 | 1.19 | 6.98 | 2181 | 1.21 | 6.81 | 6.90 |
|  | UUC | 1495 | 0.81 |  | 1420 | 0.79 |  |  |
| Leucine(L) | UUA | 1091 | 1.28 | 9.57 | 1148 | 1.29 | 10.11 | 9.84 |
|  | UUG | 1022 | 1.2 |  | 1062 | 1.19 |  |  |
|  | CUU | 1022 | 1.24 |  | 1105 | 1.24 |  |  |
|  | CUC | 649 | 0.76 |  | 647 | 0.73 |  |  |
|  | CUA | 744 | 0.88 |  | 878 | 0.99 |  |  |
|  | CUG | 536 | 0.63 |  | 507 | 0.57 |  |  |
| Isoleucine(I) | AUU | 1734 | 1.24 | 7.9 | 1754 | 1.24 | 8.16 | 8.03 |
|  | AUC | 1089 | 0.78 |  | 1088 | 0.76 |  |  |
|  | AUA | 1382 | 0.99 |  | 1477 | 1.03 |  |  |
| Methionine(M) | AUG | 833 | 1 | 1.57 | 911 | 1.00 | 1.72 | 1.65 |
| Valine(V) | GUU | 779 | 1.37 | 4.30 | 855 | 1.37 | 4.71 | 4.51 |
|  | GUC | 423 | 0.74 |  | 415 | 0.67 |  |  |
|  | GUA | 627 | 1.1 |  | 750 | 1.20 |  |  |
|  | GUG | 446 | 0.78 |  | 472 | 0.76 |  |  |
| Serine(S) | UCU | 1250 | 1.51 | 9.41 | 1218 | 1.52 | 9.12 | 9.27 |
|  | UCC | 904 | 1.09 |  | 843 | 1.05 |  |  |
|  | UCA | 993 | 1.2 |  | 1051 | 1.31 |  |  |
|  | UCG | 614 | 0.74 |  | 640 | 0.80 |  |  |
|  | AGU | 720 | 0.87 |  | 646 | 0.80 |  |  |
|  | AGC | 499 | 0.6 |  | 425 | 0.53 |  |  |
| Proline(P) | CCU | 646 | 1.02 | 4.79 | 687 | 1.04 | 4.99 | 4.89 |
|  | CCC | 647 | 1.02 |  | 660 | 1.00 |  |  |
|  | CCA | 818 | 1.29 |  | 841 | 1.27 |  |  |
|  | CCG | 423 | 0.67 |  | 452 | 0.68 |  |  |
| Threonine(T) | ACU | 677 | 1.17 | 4.38 | 678 | 1.17 | 4.38 | 4.38 |
|  | ACC | 613 | 1.06 |  | 569 | 0.98 |  |  |
|  | ACA | 663 | 1.14 |  | 729 | 1.26 |  |  |
|  | ACG | 364 | 0.63 |  | 341 | 0.59 |  |  |
| Alanine(A) | GCU | 518 | 1.35 | 2.90 | 497 | 1.23 | 2.88 | 2.89 |
|  | GCC | 376 | 0.98 |  | 391 | 0.97 |  |  |
|  | GCA | 416 | 1.08 |  | 484 | 1.20 |  |  |
|  | GCG | 225 | 0.59 |  | 240 | 0.60 |  |  |
| Tyrosine(Y) | UAU | 1484 | 1.33 | 4.16 | 1443 | 1..34 | 4.07 | 4.12 |
|  | UAC | 746 | 0.67 |  | 709 | 0.66 |  |  |
| Histidine(H) | CAU | 1069 | 1.42 | 2.85 | 1021 | 1.42 | 2.71 | 2.78 |
|  | CAC | 439 | 0.58 |  | 412 | 0.58 |  |  |
| Glutamine(Q) | CAA | 1056 | 1.36 | 2.94 | 1063 | 1.35 | 2.98 | 2.96 |
|  | CAG | 497 | 0.64 |  | 514 | 0.65 |  |  |
| Asparagine(N) | AAU | 1783 | 1.38 | 4.90 | 1687 | 1.37 | 4.64 | 4.77 |
|  | AAC | 810 | 0.62 |  | 768 | 0.63 |  |  |
| Lysine(K) | AAA | 2031 | 1.32 | 5.81 | 2106 | 1.34 | 5.95 | 5.88 |
|  | AAG | 1041 | 0.68 |  | 1044 | 0.66 |  |  |
| Aspartic acid(D) | GAU | 1190 | 1.46 | 3.08 | 1111 | 1.44 | 2.91 | 3.00 |
|  | GAC | 440 | 0.54 |  | 430 | 0.66 |  |  |
| Glutamic acid (E) | GAA | 1487 | 1.42 | 3.97 | 1386 | 1.39 | 3.77 | 3.87 |
|  | GAG | 612 | 0.58 |  | 610 | 0.61 |  |  |
| Cysteine (C) | UGU | 740 | 1.21 | 2.32 | 674 | 1.21 | 2.11 | 2.22 |
|  | UGC | 487 | 0.79 |  | 443 | 0.79 |  |  |
| Tryptophan (W) | UGG | 736 | 1 | 1.39 | 719 | 1.00 | 1.36 | 1.38 |
| Arginine (R) | CGU | 402 | 0.71 | 6.47 | 378 | 0.67 | 6.41 | 6.44 |
|  | CGC | 258 | 0.45 |  | 261 | 0.46 |  |  |
|  | CGA | 614 | 1.08 |  | 630 | 1.11 |  |  |
|  | CGG | 415 | 0.73 |  | 424 | 0.75 |  |  |
|  | AGA | 1103 | 1.93 |  | 1085 | 1.92 |  |  |
|  | AGG | 629 | 1.1 |  | 613 | 1.08 |  |  |
| Glycine(G) | GGU | 567 | 0.98 | 4.40 | 565 | 0.98 | 4.40 | 4.40 |
|  | GGC | 355 | 0.61 |  | 364 | 0.63 |  |  |
|  | GGA | 858 | 1.48 |  | 808 | 1.40 |  |  |
|  | GGG | 546 | 0.94 |  | 569 | 0.99 |  |  |
| Stop codon (*) | UAA | 1177 | 1.16 | 5.74 | 1088 | 1.08 | 5.69 | 5.72 |
|  | UAG | 850 | 0.84 |  | 858 | 0.86 |  |  |
|  | UGA | 1011 | 1 |  | 1064 | 1.06 |  |  |
|  |  |  |  |  |  |  |  |  |
| Average codons | | 52905 |  |  | 52, 909 |  |  |  |

d). *Sambucus adnata*

| **Amino acid** | **Codon** | **Count**  ***S. adnata* (ON006399)** | **RSCU** | **%RSCU** | **Count**  ***S. adnata* (ON006400)** | **RSCU** | **% RSCU** | **Average % RSCU** |
| --- | --- | --- | --- | --- | --- | --- | --- | --- |
| Phenylalanine(F) | UUU | 2100 | 1.15 | 6.88 | 2209 | 1.22 | 6.85 | 6.87 |
|  | UUC | 1538 | 0.85 |  | 1414 | 0.78 |  |  |
| Leucine(L) | UUA | 1152 | 1.29 | 10.12 | 1140 | 1.31 | 9.88 | 10.00 |
|  | UUG | 1108 | 1.24 |  | 1052 | 1.21 |  |  |
|  | CUU | 1077 | 1.21 |  | 1112 | 1.28 |  |  |
|  | CUC | 643 | 0.21 |  | 639 | 0.73 |  |  |
|  | CUA | 828 | 0.93 |  | 758 | 0.87 |  |  |
|  | CUG | 544 | 0.61 |  | 521 | 0.60 |  |  |
| Isoleucine(I) | AUU | 1744 | 1.22 | 8.13 | 1689 | 1.20 | 8.00 | 8.07 |
|  | AUC | 1108 | 0.77 |  | 1091 | 0.77 |  |  |
|  | AUA | 1443 | 1.01 |  | 1448 | 1.03 |  |  |
| Methionine(M) | AUG | 928 | 1.00 | 1.76 | 875 | 1.00 | 1.66 | 1.71 |
| Valine(V) | GUU | 769 | 1.28 | 5.54 | 805 | 1.37 | 4.44 | 4.99 |
|  | GUC | 440 | 0.73 |  | 421 | 0.72 |  |  |
|  | GUA | 722 | 1.20 |  | 717 | 1.22 |  |  |
|  | GUG | 466 | 0.78 |  | 402 | 0.69 |  |  |
| Serine(S) | UCU | 1270 | 1.51 | 9.33 | 1247 | 1.50 | 9.41 | 9.37 |
|  | UCC | 898 | 1.07 |  | 895 | 1.08 |  |  |
|  | UCA | 857 | 1.14 |  | 1014 | 1.22 |  |  |
|  | UCG | 644 | 0.77 |  | 603 | 0.73 |  |  |
|  | AGU | 758 | 0.90 |  | 712 | 0.86 |  |  |
|  | AGC | 507 | 0.60 |  | 501 | 0.60 |  |  |
| Proline(P) | CCU | 671 | 1.06 | 4.79 | 651 | 1.02 | 4.81 | 4.80 |
|  | CCC | 634 | 1.00 |  | 660 | 1.04 |  |  |
|  | CCA | 788 | 1.25 |  | 790 | 1.24 |  |  |
|  | CCG | 437 | 0.69 |  | 443 | 0.70 |  |  |
| Threonine(T) | ACU | 636 | 1.08 | 4.45 | 642 | 1.09 | 4.47 | 4.46 |
|  | ACC | 651 | 1.10 |  | 606 | 1.03 |  |  |
|  | ACA | 679 | 115 |  | 718 | 1.21 |  |  |
|  | ACG | 391 | 0.66 |  | 398 | 0.67 |  |  |
| Alanine(A) | GCU | 484 | 1.28 | 2.86 | 485 | 1.27 | 2.88 | 2.87 |
|  | GCC | 378 | 1.00 |  | 377 | 0.99 |  |  |
|  | GCA | 416 | 1.10 |  | 424 | 1.11 |  |  |
|  | GCG | 236 | 0.62 |  | 236 | 0.62 |  |  |
| Tyrosine(Y) | UAU | 1434 | 1.36 | 3.98 | 1483 | 1.35 | 4.16 | 4.07 |
|  | UAC | 668 | 0.64 |  | 717 | 0.65 |  |  |
| Histidine(H) | CAU | 1029 | 1.43 | 2.73 | 1023 | 1.41 | 2.74 | 2.74 |
|  | CAC | 412 | 0.67 |  | 426 | 0.59 |  |  |
| Glutamine(Q) | CAA | 1093 | 1.38 | 2.99 | 1130 | 1.40 | 3.05 | 3.02 |
|  | CAG | 488 | 0.62 |  | 485 | 0.60 |  |  |
| Asparagine(N) | AAU | 1780 | 1.39 | 4.85 | 1767 | 1.39 | 4.80 | 4.83 |
|  | AAC | 782 | 0.61 |  | 773 | 0.61 |  |  |
| Lysine(K) | AAA | 2063 | 1.33 | 5.86 | 1989 | 1.33 | 5.64 | 5.75 |
|  | AAG | 1033 | 0.67 |  | 994 | 0.67 |  |  |
| Aspartic acid(D) | GAU | 1234 | 1.48 | 3.16 | 1148 | 1.46 | 2.97 | 3.07 |
|  | GAC | 434 | 0.52 |  | 423 | 0.54 |  |  |
| Glutamic acid (E) | GAA | 1384 | 1.48 | 3.82 | 1444 | 1.39 | 3.94 | 3.88 |
|  | GAG | 634 | 0.63 |  | 641 | 0.61 |  |  |
| Cysteine (C) | UGU | 701 | 1.23 | 2.16 | 705 | 1.17 | 2.28 | 2.22 |
|  | UGC | 440 | 0.77 |  | 498 | 0.83 |  |  |
| Tryptophan (W) | UGG | 713 | 1.00 | 1.35 | 724 | 1.00 | 1.37 | 1.36 |
| Arginine (R) | CGU | 368 | 0.66 | 6.35 | 397 | 0.70 | 6.40 | 6.38 |
|  | CGC | 264 | 0.47 |  | 270 | 0.48 |  |  |
|  | CGA | 586 | 1.05 |  | 620 | 1.10 |  |  |
|  | CGG | 420 | 0.75 |  | 413 | 0.73 |  |  |
|  | AGA | 1095 | 1.96 |  | 1077 | 1.91 |  |  |
|  | AGG | 624 | 1.12 |  | 604 | 1.07 |  |  |
| Glycine(G) | GGU | 535 | 0.94 | 4.31 | 555 | 0.95 | 4.43 | 4.37 |
|  | GGC | 366 | 0.64 |  | 381 | 0.65 |  |  |
|  | GGA | 820 | 1.44 |  | 858 | 1.64 |  |  |
|  | GGG | 555 | 0.98 |  | 550 | 0.94 |  |  |
| Stop codon (*) | UAA | 1107 | 1.16 | 5.41 | 1156 | 1.13 | 5.81 | 5.61 |
|  | UAG | 792 | 0.83 |  | 863 | 0.84 |  |  |
|  | UGA | 958 | 1.01 |  | 1055 | 1.03 |  |  |
|  |  |  |  |  |  |  |  |  |
| Average codons | | 52, 857 |  |  | 52, 865 |  |  |  |
