# Supplementary material for: Comparative chloroplast genome analysis of Sambucus L. (Viburnaceae): inference for phylogenetic relationships among the closely related Sambucus adnata Wall. ex DC Sambucus javanica Blume
Source: Front Plant Sci. 2023 Jun 16;14:1179510. doi: 10.3389/fpls.2023.1179510 (PMC10313135; doi:10.3389/fpls.2023.1179510)
Supplement: Supplementary file 9 [file Table_7.docx]

Supplementary Material

**Table S6** Non-synonymous and synonymous substitution rates

|  | Method | Sw.Ka | Sc.Ka | Sc.Ks | Sw.Ks | Sw.Ka/Ks | Sc.Ka/Ks |  |
| --- | --- | --- | --- | --- | --- | --- | --- | --- |
| rpl2 | MS | 0.960515 | 0.960515 | 1.13523 | 1.13523 | 0.846096 | 0.846096 | IR |
| rpl2 | MA | 0.995961 | 0.959614 | 1.12843 | 1.12843 | 0.850396 | 0.850396 | IR |
| rps7 | MS | 0.906186 | 0.906186 | 1.38535 | 1.38535 | 0.654122 | 0.654122 | IR |
| rps7 | MA | 0.90511 | 0.90511 | 1.40288 | 1.40288 | 0.645182 | 0.645182 | IR |
| rpl23 | MS | 0.971233 | 0.971233 | 1.11735 | 1.11735 | 0.86923 | 0.86923 | IR |
| rpl23 | MA | 0.971309 | 0.971309 | 1.11715 | 1.11715 | 0.869452 | 0.869452 | IR |
| rps12 | MS | 0.984626 | 0.984626 | 1.05585 | 1.05585 | 0.932543 | 0.932543 | IR |
| rps12 | MA | 0.983443 | 0.983443 | 1.06257 | 1.06257 | 0.925532 | 0.925532 | IR |
| ycf2 | MS | 0.992993 | 0.992908 | 1.03104 | 1.03062 | 0.963492 | 0.963021 | IR |
| ycf2 | MA | 0.99151 | 0.99139 | 1.03772 | 1.03714 | 0.095996 | 0.955356 | IR |
| ycf15 | MS | 1.10246 | 1.10246 | 0.636586 | 0.636586 | 1.73183 | 1.73183 | IR |
| ycf15 | MA | 1.09454 | 1.09454 | 0.633636 | 0.633636 | 1.7274 | 1.7274 | IR |
| accD | MS | 0.965507 | 0.964459 | 1.20697 | 1.19844 | 0.805638 | 0.799075 | LSC |
| accD | MA | 0.96826 | 0.967067 | 1.18347 | 1.1746 | 0.824334 | 0.817144 | LSC |
| atpA | MS | 0.952126 | 0.961376 | 1.13929 | 1.17498 | 0.810333 | 0.843841 | LSC |
| atpA | MA | 0.951977 | 0.961151 | 1.13932 | 1.17461 | 0.810462 | 0.843615 | LSC |
| atpE | MS | 0.967836 | 0.972368 | 1.09929 | 1.11889 | 0.864994 | 0.88454 | LSC |
| atpE | MA | 0.972958 | 0.97707 | 1.08559 | 1.10411 | 0.881213 | 0.900038 | LSC |
| atpF | MS | 1.05952 | 1.06455 | 0.770584 | 0.78673 | 1.34674 | 1.38148 | LSC |
| atpF | MA | 1.06258 | 1.06551 | 0.763822 | 0.773278 | 1.37413 | 1.39497 | LSC |
| atpH | MS | 1.03189 | 0.992303 | 1.02945 | 0.924816 | 1.11578 | 0.963915 | LSC |
| atpH | MA | 1.03266 | 0.996238 | 1.01433 | 0.922007 | 1.12001 | 0.982168 | LSC |
| atpI | MS | 1.00578 | 1.01952 | 0.930786 | 0.979369 | 1.02697 | 1.09534 | LSC |
| atpI | MA | 1.0037 | 1.01685 | 0.938468 | 0.986461 | 1.01747 | 1.08353 | LSC |
| cemA | MS | 0.969996 | 0.971811 | 1.12343 | 1.1324 | 0.856587 | 0.865039 | LSC |
| cemA | MA | 0.970092 | 0.972728 | 1.11497 | 1.1275 | 0.860393 | 0.872423 | LSC |
| clpP | MS | 1.0019 | 1.0019 | 0.993888 | 0.993888 | 1.00807 | 1.00807 | LSC |
| clpP | MA | 1.00218 | 1.00218 | 0.992991 | 0.992991 | 1.00926 | 1.00926 | LSC |
| infA | MS | 0.922344 | 0.922344 | 1.35771 | 1.35771 | 0.679337 | 0.679337 | LSC |
| infA | MA | 0.92931 | 0.92931 | 1.31263 | 1.31263 | 0.707978 | 0.707978 | LSC |
| ndhJ | MS | 0.988547 | 0.988547 | 1.03869 | 1.03869 | 0.951722 | 0.951722 | LSC |
| ndhJ | MA | 0.982851 | 0.982851 | 1.06099 | 1.06099 | 0.92635 | 0.92635 | LSC |
| ndhK | MS | 0.972237 | 0.969617 | 1.1159 | 1.10874 | 0.876886 | 0.868911 | LSC |
| ndhK | MA | 0.972337 | 0.969857 | 1.11591 | 1.10931 | 0.876525 | 0.869116 | LSC |
| petB | MS | 0.990741 | 0.990741 | 1.03222 | 1.03222 | 0.95982 | 0.95982 | LSC |
| petB | MA | 0.9911 | 0.9911 | 1.03089 | 1.03089 | 0.961404 | 0.961404 | LSC |
| petD | MS | 0.95492 | 0.957025 | 1.15634 | 1.16333 | 0.820853 | 0.827635 | LSC |
| petD | MA | 0.962078 | 0.96132 | 1.13595 | 1.13224 | 0.849713 | 0.846268 | LSC |
| petG | MS | 1.11467 | 1.11467 | 0.650713 | 0.650713 | 1.713 | 1.713 | LSC |
| petG | MA | 1.11482 | 1.11482 | 0.649002 | 0.649002 | 1.71775 | 1.71775 | LSC |
| petL | MS | 0.998045 | 0.975303 | 1.07538 | 1.00537 | 0.992712 | 0.906935 | LSC |
| petL | MA | 0.995879 | 0.974827 | 1.08333 | 1.01197 | 0.984103 | 0.899846 | LSC |
| petN | MS | 1.21484 | 1.21484 | 0.557665 | 0.557665 | 2.17845 | 2.17845 | LSC |
| petN | MA | 1.09946 | 1.09946 | 0.508514 | 0.508514 | 2.1621 | 2.1621 | LSC |
| psaB | MS | 1.01846 | 1.02143 | 0.924834 | 0.934853 | 1.08944 | 1.10444 | LSC |
| psaB | MA | 1.02102 | 1.02378 | 0.914806 | 0.924522 | 1.10437 | 1.11912 | LSC |
| psaI | MS | 1.08121 | 1.08121 | 0.684336 | 0.684336 | 1.57995 | 1.57995 | LSC |
| psaI | MA | 1.09289 | 1.09289 | 0.686529 | 0.689529 | 1.58498 | 1.58498 | LSC |
| psaJ | MS | 1.00186 | 0.984115 | 1.04094 | 0.995465 | 1.00642 | 0.945409 | LSC |
| psaJ | MA | 0.998114 | 0.982096 | 1.04491 | 1.00448 | 0.993661 | 0.939886 | LSC |
| psbA | MS | 1.00522 | 0.99912 | 1.00031 | 0.981472 | 1.0242 | 0.999606 | LSC |
| psbA | MA | 1.00156 | 0.996501 | 1.01264 | 0.994268 | 1.00733 | 0.98405 | LSC |
| psbC | MS | 1.0397 | 1.04129 | 0.856537 | 0.879492 | 1.18216 | 1.2157 | LSC |
| psbC | MA | 1.0388 | 1.04411 | 0.848658 | 0.878806 | 1.18205 | 1.23031 | LSC |
| psbD | MS | 0.976207 | 0.976536 | 1.08339 | 1.08473 | 0.89995 | 0.901369 | LSC |
| psbD | MA | 0.978074 | 0.978546 | 1.0739 | 1.07574 | 0.909208 | 0.911209 | LSC |
| psbE | MS | 0.905746 | 0.905746 | 1.33097 | 1.33097 | 0.680515 | 0.680515 | LSC |
| psbE | MA | 0.907944 | 0.907944 | 1.30843 | 1.30843 | 0.693919 | 0.693919 | LSC |
| psbF | MS | 1.01138 | 1.01138 | 0.972783 | 0.972783 | 1.03968 | 1.03968 | LSC |
| psbF | MA | 1.01086 | 1.01086 | 0.974803 | 0.974803 | 1.03699 | 1.039167 | LSC |
| psbH | MS | 0.921833 | 0.926906 | 1.29196 | 1.32141 | 0.697612 | 0.71744 | LSC |
| psbH | MA | 0.925454 | 0.928899 | 1.28552 | 1.30913 | 0.706922 | 1.28552 | LSC |
| psbI | MS | 1.14509 | 1.14509 | 0.686882 | 0.686882 | 1.66709 | 1.66709 | LSC |
| psbI | MA | 1.15549 | 1.15549 | 0.691967 | 0.66986 | 1.66986 | 1.66986 | LSC |
| psbK | MS | 0.928174 | 1.00553 | 0.979957 | 1.20065 | 0.773061 | 1.02609 | LSC |
| psbK | MA | 0.921137 | 1.00471 | 0.98469 | 1.20065 | 0.773061 | 1.02034 | LSC |
| psbL | MS | 1.00553 | 1.02282 | 0.90308 | 0.979957 | 1.02609 | 1.1326 | LSC |
| psbL | MA | 1.00471 | 1.02509 | 0.891673 | 0.98469 | 1.02609 | 1.14963 | LSC |
| psbM | MS | 1.02282 | 0.839653 | 1.62112 | 0.90308 | 1.1326 | 0.517945 | LSC |
| psbM | MA | 1.02509 | 0.844118 | 1.61563 | 0.891673 | 1.14963 | 0.522468 | LSC |
| psbN | MS | 0.839653 | 0.942854 | 1.17454 | 1.62112 | 0.517945 | 0.802745 | LSC |
| psbN | MA | 0.844118 | 0.944579 | 1.19453 | 1.61563 | 0.522468 | 0.790757 | LSC |
| psbT | MS | 0.942854 | 0.945439 | 1.15765 | 1.17454 | 0.802745 | 0.816685 | LSC |
| psbT | MA | 0.944579 | 0.950127 | 1.17174 | 1.194553 | 0.790757 | 0.824948 | LSC |
| psbZ | MS | 0.945439 | 0.915995 | 1.32272 | 1.15765 | 0.816685 | 0.692508 | LSC |
| psbZ | MA | 0.950127 | 0.944278 | 1.35096 | 1.15174 | 0.824948 | 0.698968 | LSC |
| rbcL | MS | 0.990966 | 0.987455 | 1.04505 | 1.03226 | 0.960001 | 0.944891 | LSC |
| rbcL | MA | 0.991756 | 0.988097 | 1.04352 | 1.02997 | 0.962896 | 0.946888 | LSC |
| rpl16 | MS | 0.992794 | 0.992794 | 1.02793 | 1.02793 | 0.965822 | 0.965822 | LSC |
| rpl16 | MA | 0.990616 | 0.990616 | 1.03659 | 1.03659 | 0.955651 | 0.955651 | LSC |
| rpl22 | MS | 1.00202 | 1.01913 | 0.91382 | 0.990901 | 1.01122 | 1.11524 | LSC |
| rpl22 | MA | 1.00016 | 1.01697 | 0.919529 | 0.999232 | 1.00093 | 1.10596 | LSC |
| rpl36 | MS | 1.04084 | 1.04084 | 0.838298 | 0.838298 | 1.24161 | 1.24161 | LSC |
| rpl36 | MA | 1.03846 | 1.03846 | 0.85428 | 0.85428 | 1.2156 | 1.2156 | LSC |
| rpoA | MS | 0.928853 | 0.925581 | 1.30867 | 1.29722 | 0.716036 | 0.707268 | LSC |
| rpoA | MA | 0.92855 | 0.925395 | 1.31208 | 1.30187 | 1.30187 | 1.31208 | LSC |
| rpoB | MS | 0.962512 | 0.959581 | 1.15056 | 1.14046 | 0.843964 | 0.834009 | LSC |
| rpoB | MA | 0.960274 | 0.957652 | 1.15561 | 1.14735 | 0.836952 | 0.828701 | LSC |
| rpoC1 | MS | 0.939198 | 0.938214 | 1.24912 | 1.24425 | 0.754829 | 0.7511 | LSC |
| rpoC1 | MA | 0.939705 | 0.938583 | 1.24519 | 1.23936 | 0.758218 | 0.753768 | LSC |
| rpoC2 | MS | 0.973909 | 0.979104 | 1.13154 | 1.11071 | 0.876835 | 0.856449 | LSC |
| rpoC2 | MA | 0.976901 | 0.972474 | 1.11598 | 1.09695 | 0.890561 | 1.11598 | LSC |
| rps4 | MS | 0.969733 | 0.969733 | 1.11606 | 1.11606 | 0.868892 | 0.072932 | LSC |
| rps4 | MA | 0.967054 | 0.967054 | 1.13364 | 1.13364 | 0.85305 | 0.85305 | LSC |
| rps8 | MS | 0.958693 | 0.958683 | 1.15043 | 1.15043 | 0.833334 | 0.099783 | LSC |
| rps8 | MA | 0.956586 | 0.956586 | 1.15885 | 1.15885 | 0.82546 | 0.82546 | LSC |
| rps11 | MS | 1.00656 | 1.00656 | 0.980505 | 0.980505 | 1.02657 | 1.02657 | LSC |
| rps11 | MA | 1.00635 | 1.00635 | 0.981093 | 0.981093 | 1.02575 | 1.02575 | LSC |
| rps18 | MS | 0.874714 | 0.874714 | 1.55401 | 1.55401 | 0.562874 | 0.562874 | LSC |
| rps18 | MA | 0.878265 | 0.878265 | 1.55707 | 1.55707 | 0.56405 | 0.56405 | LSC |
| ycf3 | MS | 0.902345 | 0.889261 | 1.41094 | 1.40433 | 0.642547 | 0.637351 | LSC |
| ycf3 | MA | 0.897242 | 0.894504 | 1.45629 | 1.45122 | 0.618269 | 0.614234 | LSC |
| ycf4 | MS | 0.983217 | 0.983217 | 1.05687 | 1.05687 | 0.939309 | 0.930309 | LSC |
| ycf4 | MA | 0.984421 | 0.984421 | 1.05286 | 1.05286 | 0.934994 | 0.934994 | LSC |
| ccsA | MS | 0.951985 | 0.956409 | 1.1842 | 1.20302 | 0.791326 | 0.807643 | SCC |
| ccsA | MA | 0.957685 | 0.962181 | 1.16542 | 1.18517 | 0.80806 | 0.825606 | SCC |
| ndhA | MS | 0.956487 | 0.956523 | 1.16608 | 1.16775 | 0.819082 | 0.820287 | SCC |
| ndhA | MA | 0.964882 | 0.964745 | 1.13115 | 1.13199 | 0.852377 | 0.852889 | SCC |
| ndhD | MS | 1.01186 | 1.01174 | 0.960793 | 0.960004 | 1.05402 | 1.05303 | SCC |
| ndhD | MA | 1.01201 | 1.01194 | 0.960649 | 0.959892 | 1.0543 | 1.05339 | SCC |
| ndhE | MS | 0.933708 | 0.932442 | 1.29137 | 1.29285 | 0.722208 | 0.722057 | SCC |
| ndhE | MA | 0.933688 | 0.932604 | 1.29311 | 1.29484 | 0.721081 | 0.721213 | SCC |
| ndhG | MS | 0.918991 | 0.918991 | 1.37678 | 1.37678 | 0.667493 | 0.667493 | SCC |
| ndhG | MA | 0.930119 | 0.930119 | 1.3213 | 1.3213 | 0.70394 | 0.70394 | SCC |
| ndhH | MS | 0.992398 | 0.992303 | 1.02945 | 1.02925 | 0.964198 | 0.963915 | SCC |
| ndhH | MA | 0.996062 | 0.996238 | 1.01433 | 1.01505 | 0.981298 | 0.982168 | SCC |
| ndhI | MS | 0.966329 | 0.97158 | 1.12194 | 1.14691 | 0.84255 | 0.865983 | SCC |
| ndhI | MA | 0.965929 | 0.970908 | 1.12477 | 1.14947 | 0.840324 | 0.863207 | SCC |
| psaC | MS | 1.04129 | 1.03857 | 0.882573 | 0.856537 | 1.2157 | 1.17675 | SCC |
| psaC | MA | 1.04411 | 1.03766 | 0.881811 | 0.848658 | 1.23031 | 1.17674 | SCC |
| ycf1 | MS | 1.0196 | 1.01885 | 0.931776 | 0.927955 | 1.09876 | 1.09345 | SCC |
| ycf1 | MA | 1.02248 | 1.02126 | 0.92566 | 0.919579 | 1.09876 | 1.10328 | SCC |
